# Supplementary material for: Overlooked and widespread pennate diatom-diazotroph symbioses in the sea
Source: Nat Commun. 2022 Feb 10;13:799. doi: 10.1038/s41467-022-28065-6 (PMC8831587; doi:10.1038/s41467-022-28065-6)
Supplement: Supplementary file 1 — Supplementary Information [file 41467_2022_28065_MOESM1_ESM.pdf]

Supplementary Information

Overlooked and widespread pennate diatom-diazotroph symbioses in the sea

Schvarcz, C. R., Wilson, S. T., Caffin, M., Stancheva, R., Li, Q., Turk-Kubo, K. A., White, A. E., Karl, D. M., Zehr, J. P. & Steward G. F.
https://doi.org/10.1038/s41467-022-28065-6

Inventory of Supplementary Information

Supplementary Notes

Supplementary Note 1. Detailed taxonomic description of *Epithemia pelagica sp*. nov.
Supplementary Note 2. Detailed taxonomic description of *Epithemia catenata sp*. nov.

Supplementary Figures

Supplementary Fig. 1: *E. pelagica*, light and scanning electron micrographs of both uncleaned and hydrogen peroxide-cleaned frustules.
Supplementary Fig. 2: *E. pelagica*, scanning electron micrographs of hydrogen peroxide-cleaned frustules.
Supplementary Fig. 3: *E. catenata*, light micrographs of hydrogen peroxide-cleaned frustules.
Supplementary Fig. 4: *E. catenata*, scanning electron micrographs of uncleaned and hydrogen peroxide-cleaned frustules.
Supplementary Fig. 5: *E. catenata*, transmission electron micrographs of hydrogen peroxide-cleaned frustules.
Supplementary Fig. 6: *E. pelagica* UHM3200, light micrographs of live cells.
Supplementary Fig. 7: *E. pelagica* UHM3201, light micrographs of live cells.
Supplementary Fig. 8: *E. pelagica* UHM3202, light micrographs of live cells.
Supplementary Fig. 9: *E. pelagica* UHM3203, light micrographs of live cells.
Supplementary Fig. 10: *E. pelagica* UHM3204, light micrographs of live cells.
Supplementary Fig. 11: *E. catenata* UHM3210, light micrographs of live cells.
Supplementary Fig. 12: *E. catenata* UHM3211, light micrographs of live cells.
Supplementary Fig. 13: Phylogenetic analysis of *E. pelagica* and *E. catenata*, including diverse representative taxa from the diatom orders Surirellales and Rhopalodiales, based on the small subunit ribosomal RNA gene (SSU/18S), aligned de novo using MAFFT.
Supplementary Fig. 14: Phylogenetic analysis of *E. pelagica* and *E. catenata*, including diverse representative taxa from the diatom orders Surirellales and Rhopalodiales, based on the small subunit ribosomal RNA gene (SSU/18S), aligned according to the global SILVA alignment for SSU genes using SINA.
Supplementary Fig. 15: Phylogenetic analysis of *E. pelagica* and *E. catenata*, including diverse representative taxa from the diatom orders Surirellales and Rhopalodiales, based on the large subunit ribosomal RNA gene (LSU/28S), aligned de novo using MAFFT.
Supplementary Fig. 16: Phylogenetic analysis of *E. pelagica* and *E. catenata*, including diverse representative taxa from the diatom orders Surirellales and Rhopalodiales, based on the large subunit ribosomal RNA gene (LSU/28S), aligned according to the global SILVA alignment for LSU genes using SINA.
Supplementary Fig. 17: Phylogenetic analysis of *E. pelagica* and *E. catenata*, including diverse representative taxa from the diatom orders Surirellales and Rhopalodiales, based on the apocytochrome b gene (*cob*).

Supplementary Fig. 18: Phylogenetic analysis of *E. pelagica* and *E. catenata*, including diverse representative taxa from the diatom orders Surirellales and Rhopalodiales, based on the photosystem II CP43 reaction center protein gene (*psbC*).
Supplementary Fig. 19: Phylogenetic analysis of *E. pelagica* and *E. catenata*, including diverse representative taxa from the diatom orders Surirellales and Rhopalodiales, based on the ribulose-bisphosphate carboxylase gene (*rbcL*).
Supplementary Fig. 20: Light micrographs and PCR results demonstrating the loss of endosymbionts from *Epithemia* cultures, after extended propagation on nitrogen-replete medium.
Supplementary Fig. 21: Two maps consisting of (a) a map of environmental *nifH* sequence hits to *E. pelagica*, with less stringent percent identity cutoffs and (b) a map of environmental *nifH* sequence hits to *E. catenata*.
Supplementary Fig. 22: Observations of *E. pelagica* symbioses at Station ALOHA, including (a) quantitative PCR measurements of *E. pelagica* (LSU gene) and *Ep*SB (*nifH* gene) in water column samples and (b) the interannual detection of *Ep*SB *nifH* sequences in metagenomes constructed from deep sediment trap material.
Supplementary Fig. 23: Phylogeny of previously published environmental *nifH* sequences in the NCBI non-redundant nucleotide (nt) database that share >95% nucleotide identity with *Ep*SB and *Ec*SB.
Supplementary Fig. 24. Nucleotide alignment of *Epithemia nifH* sequences with previously published UCYN-C primers and probes.

Supplementary Tables

Supplementary Table 1: Metadata and NCBI accession numbers for all *E. pelagica* and *E. catenata* isolates.
Supplementary Table 2: Key of *Epithemia* strains used in each set of micrograph images.
Supplementary Table 3: SH and AU test results for different topological constraints on *E. catenata*.

Supplementary References

Supplementary Notes

Supplementary Note 1. Detailed taxonomic description of *Epithemia pelagica*.

***Epithemia pelagica* Schvarcz, Stancheva & Steward sp. nov.**

Figures (1a–c, n)

Supplementary Figures (1a–n, 2a–h, 6–10)

Description

Observation by LM of cells with protoplast: Cells solitary, arcuate, usually lying in valve view (Supplementary Figs. 6–10). Each cell contains a large plate-like golden-brown chloroplast with lobed margins (Supplementary Figs. 6–10) and usually one or two centrally located spherical or slightly elongated colorless cyanobacterial endosymbionts (Fig. 1b, c, n).

Observation by LM of frustules: Frustules small, 6.7–17.8 µm long, 5–9.8 µm wide, strongly dorsiventral (Supplementary Fig. 1a–h). Valves lunate with rounded apices, dorsal margin convex, ventral margin concave. Raphe-bearing sternum positioned on the dorsal margin, slightly bent down towards the dorsal margin at the center of the valve (Supplementary Fig. 1b, e, g). Transapical costae fine, 28–33 in 10 µm, better resolved near by the raphe sternum, where 4 to 10 costae are thicker (Supplementary Fig. 1a–h). Striae not resolvable with LM.

Observation by SEM of frustules: Frustules like orange-segments, with strongly lunate valves and girdles wider dorsally than ventrally (Supplementary Fig. 1a, j). Externally, valve face slightly undulate—concave along the keel and ventral mantle, convex in the middle portion of the valve (Fig. 1a; Supplementary Fig. 1k, l). The valve face without warts or other ornamentation (Supplementary Fig. 1i, k). Externally, raphe-bearing sternum pronounced, rising from the valve apices, running towards the dorsal margin and bent down towards the dorsal margin at the center of the valve (Supplementary Fig. 1i–k). Central fissures of the raphe bordered by siliceous flanges (Supplementary Figs. 1i, k; 2a, c). Raphe polar ends simple, curved around the valve apices and ending in slight thickening (Supplementary Fig. 2e). Costae running from margin to margin transapically, becoming slightly wider and thicker near the raphe sternum (Supplementary Fig. 1i, k, l). Striae alternated by costae (Supplementary Fig. 2a, c, e). Striae uniseriate, composed of areolae with complicated structure (Supplementary Fig. 2a, c, e). Areolae occluded externally by a flap-like occlusion, opened by two narrow crescent-shaped slits on both sides toward the costae, forming two clearly visible transapical rows of openings

between costae (Supplementary Fig. 2a, c, e). Copulae many (Supplementary Fig. 1j, l), both valvocopulae and copulae ribbed and areolated (Supplementary Fig. 2g).

The internal view shows delicate costae running from margin to margin, continuing across the raphe where they are thicker in the form of transverse ridges, more pronounced near the center and poles of the valve (Supplementary Figs. 1m, n; 2b, d, f). One or two central costae may be shortened, not reaching the ventral side (Supplementary Fig. 1m, n). The proximal raphe not visible (Supplementary Fig. 2b). The distal raphe ends at the valve poles beyond the last thickened costa (Supplementary Fig. 2f). Two rows of slit openings visible between costae, forming a single uniseriate stria (Supplementary Fig. 2d–h).

Reference culture: This novel species has been described and illustrated from cultures UHM3200 and UHM3201. Strain *Epithemia pelagica* UHM3201 is stored at the Center for Microbial Oceanography: Research and Education (C-MORE) at the University of Hawai'i at Mānoa, in Honolulu, HI, USA.

Additional strains: *Epithemia pelagica* UHM3202, UHM3203, UHM3204.

All strains are genetically similar, with pairwise sequence alignments having percent identities greater than 99.3, 99.2, 99.4, 99.8, and 99.8 for the genes small subunit ribosomal RNA (18S; 1,732 bp), large subunit ribosomal RNA (28S; 500 bp), apocytochrome b (*cob*; 694 bp), photosystem II CP43 reaction center protein (*psbC*; 1,207 bp), and ribulose-bisphosphate carboxylase (*rbcL*; 1,492 bp), respectively.

Supplementary Note 2. Detailed taxonomic description of *Epithemia catenata*.

***Epithemia catenata* Schvarcz, Stancheva & Steward sp. nov.**

Figures (1d–g, o)

Supplementary Figures (3a–c, 4a–k, 5a–f, 11, 12)

Description

Observation by LM of cells with protoplast: Cells joined together in chains, straight or curved, containing up to 60–70 cells (Fig. 1g). Cells connected by their valves in chains visible in girdle view (Fig. 1e). Girdles wider than valves, appear slightly rhomboidal (Fig. 1e, f; Supplementary Figs. 11, 12). Each cell contains a large axial plate-like golden-brown chloroplast (Fig. 1e, f, Supplementary Figs. 11, 12) and usually one or two centrally located spherical or slightly elongated colorless cyanobacterial endosymbionts (Fig. 1e, f, o; Supplementary Figs. 11, 12).

Observation by LM of frustules: Valves elliptical with raphe system central or nearly so, continuing from pole to pole, slightly sigmoid, keeled, and fibulate (Supplementary Fig. 3a–c). Frustules delicate, translucent without visible striation. Frustules 17.2–28.8 µm along apical axis, 8.2–13.7 µm along transapical axis, 11.7–16.9 µm along pervalvar axis, fibulae 8–12 in 10 µm, 2–3 µm long.

Observation by SEM of frustules: Frustules with elliptical valves (Supplementary Fig. 4g–i) and wider slightly rhomboidal girdles (Supplementary Fig. 4a–f). The overlap between epi-, and hypocingulum is visible on some girdles (Supplementary Fig. 4b), but copulae are not resolvable. Valves elliptical, symmetrical about the apical axis with flat uneven surface (Supplementary Fig. 4c, d, f, g) and high narrow raphe keel with axial position. The keel height decreases at the center of the valve and towards the poles (Supplementary Fig. 4b–g). The valves of neighboring frustules are connected to each other and keel is involved in cell–cell interlock (Supplementary Fig. 4e). Raphe central node forms gap in the fibulae between both raphe branches (Supplementary Fig. 4h, i). Fibulae elongated, without additional structures supporting the keel (Supplementary Fig. 4j, k). Valve perforations (such as pores and areolae) not resolvable with SEM in both uncleaned (Supplementary Fig. 4a–g) and hydrogen peroxide-cleaned valves (Supplementary Fig. 4h, i). During the chemical treatment (hydrogen peroxide cleaning of organics) chains and frustules disintegrated and delicate valves collapsed (Supplementary Fig. 4h–k), visible in LM only as translucent valves with fibulate keel (Supplementary Fig. 3a–c). Bacteria were attached to organic coating of uncleaned frustules (Supplementary Fig. 4 a–g).

Observation by TEM of frustules: Hydrogen peroxide-cleaned valves have been observed with TEM. Valves elliptical with distinct axial slightly sigmoid keel (Supplementary Fig. 5a, c). Raphe with long fibulae (Supplementary Fig. 5b, d). Raphe branches separated by a central node (Supplementary Fig. 5a–c), and distal raphe ends with branching structure (Supplementary Fig. 5e, f). Microfibrils with unknown chemical composition were the only visible structural components of the valve walls (Supplementary Fig. 5g), but it is not clear if they were associated with the surface of the silica (and secreted from the cell) or incorporated within it (Supplementary Fig. 5b, c, e, g).
Reference culture: This novel species has been described from culture UHM3210. Strain *Epithemia catenata* UHM3210 is stored at the Center for Microbial Oceanography: Research and Education (C-MORE) at the University of Hawai'i at Mānoa, in Honolulu, HI, USA.
Additional strains: *Epithemia catenata* UHM3211.
Both strains are genetically similar, with pairwise sequence alignments having percent identities of 99.6, 99.2, 99.7, 100, and 100 for the genes small subunit ribosomal RNA (18S; 1,732 bp), large subunit ribosomal RNA (28S; 503 bp), apocytochrome b (*cob*; 694 bp), photosystem II CP43 reaction center protein (*psbC*; 1,207 bp), and ribulose-bisphosphate carboxylase (*rbcL*; 1,491 bp), respectively.

Supplementary Figures

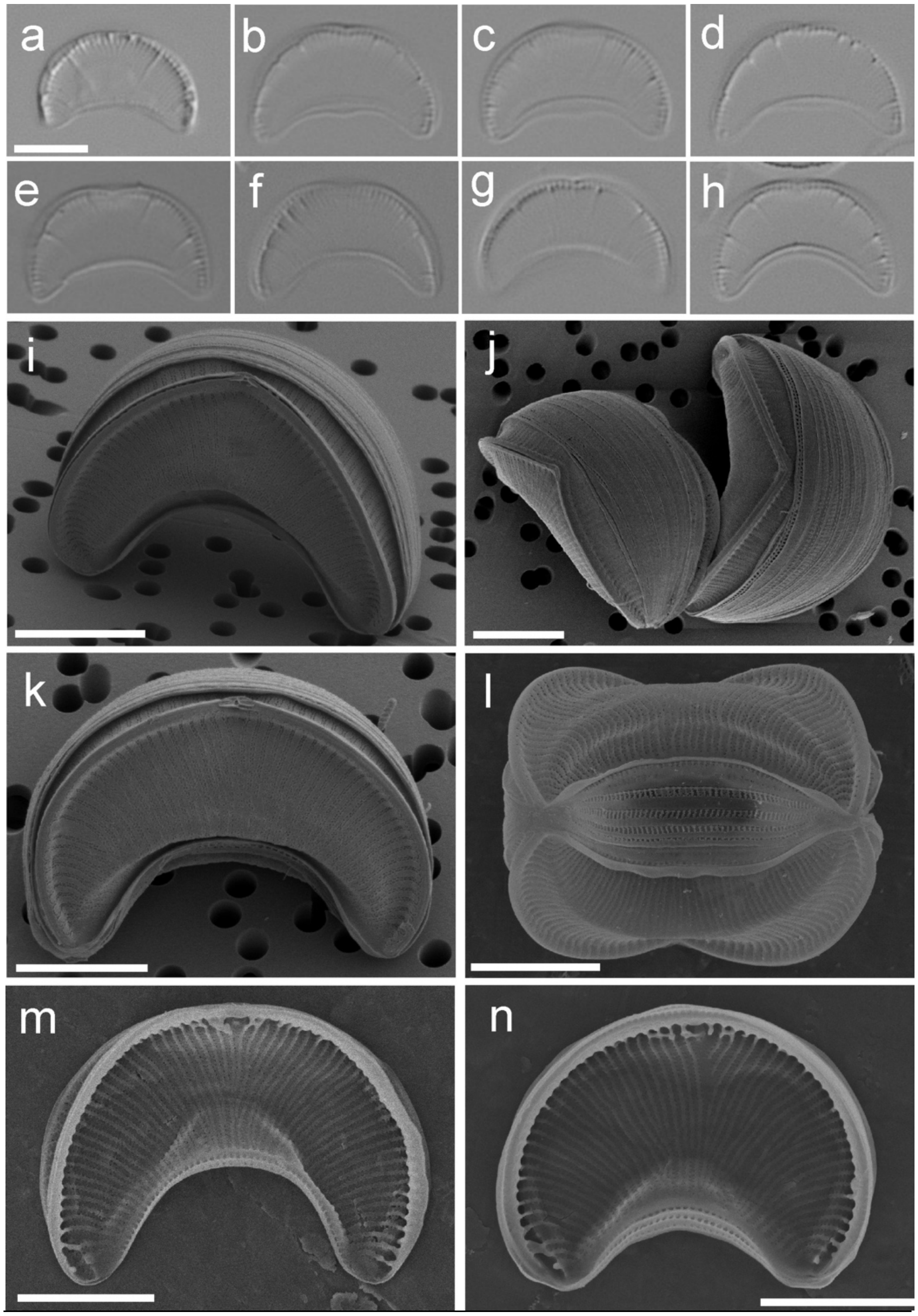

Supplementary Fig. 1. *Epithemia pelagica* sp. nov. LM (**a–h**) and SEM (**i–n**) micrographs. **a–h**, valve views; **i–l**, external frustules views, showing valve face (**i**, **k**), dorsal girdle (**j**) and ventral girdle (**l**); **m–n**, interval valve views. **b** presents the holotype. All images from hydrogen peroxide-cleaned frustules, except for **i**, **j**, **k**. Scale bars are 5 µm.

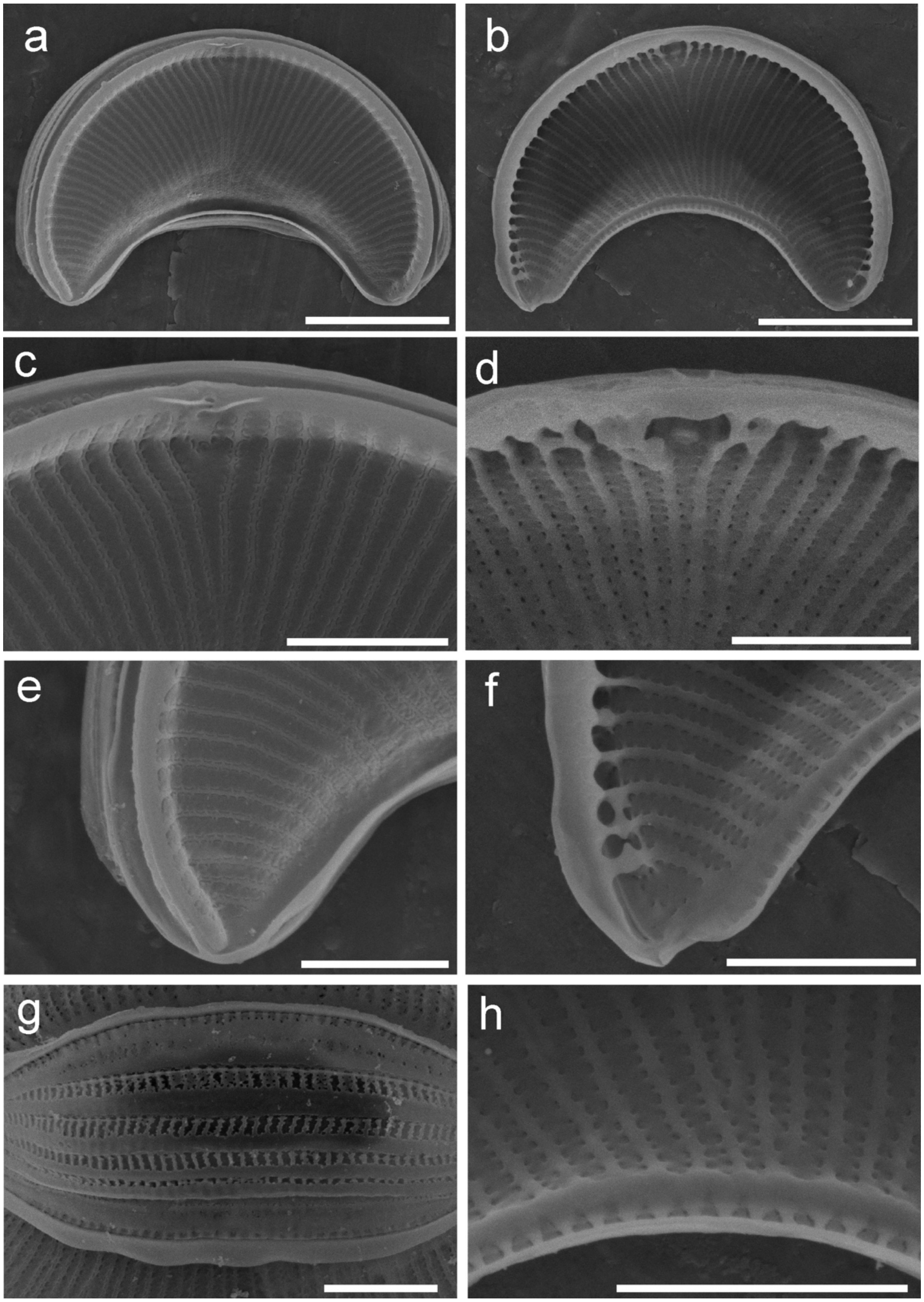

Supplementary Fig. 2. *Epithemia pelagica* sp. nov. SEM micrographs. **a**, **c**, **e**, showing external details from the same valve; **b**, **d**, **f**, **h**, showing internal details from the same valve; **g**, external view of areolate ventral copulae. All images from hydrogen peroxide-cleaned frustules. Scale bars are 5 μm (**a**, **b**) and 2 μm (**c–h**).

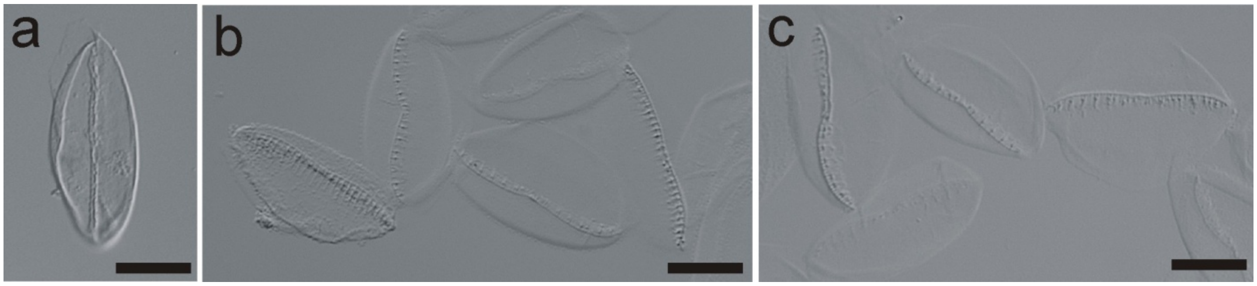

Supplementary Fig. 3. *Epithemia catenata* sp. nov. LM micrographs. **a–c**, translucent valves with distinct axial keeled raphe. **a** presents the holotype. All images from hydrogen peroxide-cleaned frustules. Scale bars are 10 µm.

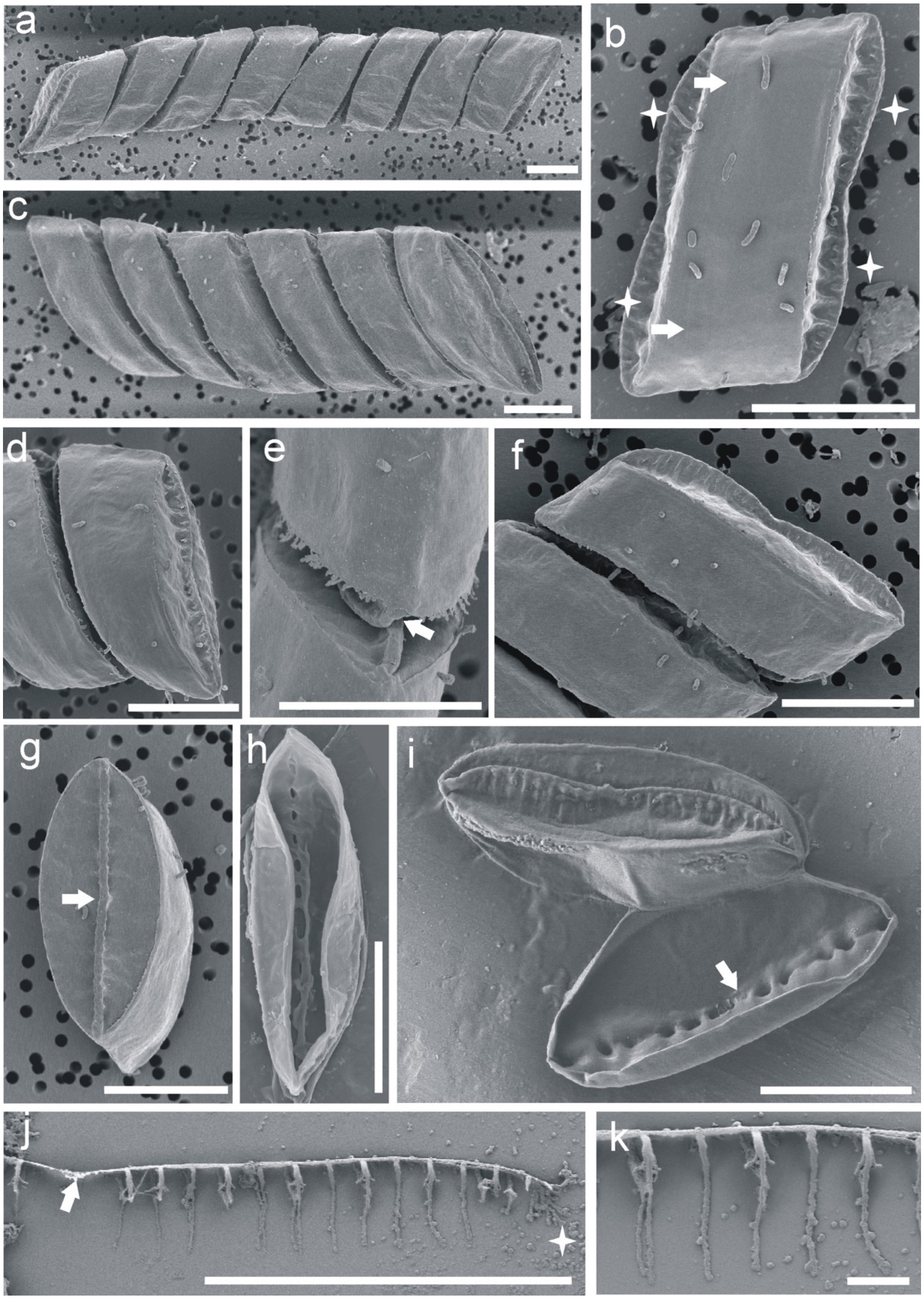

Supplementary Fig. 4. *Epithemia catenata* sp. nov. SEM micrographs.

a, **c**, **d**, **e**, **f**, girdle views of cells connected in chains; **b**, single frustule in girdle view—arrows show the overlap between both cingula, stars indicate the branches of the keeled raphe. **e**,

connection between two frustules by interlocking raphe keels of each valve (arrow). **g**, single frustule in valve view—note the axial position of the raphe keel and the central node (arrow). **h**, **i**, interval valve views (arrow). **j**, disintegrated frustule showing raphe central node (arrow) and one of the raphe branches with fibulae and branched distal end (star). **k**, element of fibulae. Images **a–g** are from cells with protoplast, and bacteria are visible on the surface of diatom cells. Images **h–k** are from hydrogen peroxide-cleaned frustules. Scale bars are 10 µm (**a–j**) and 1 µm (**k**).

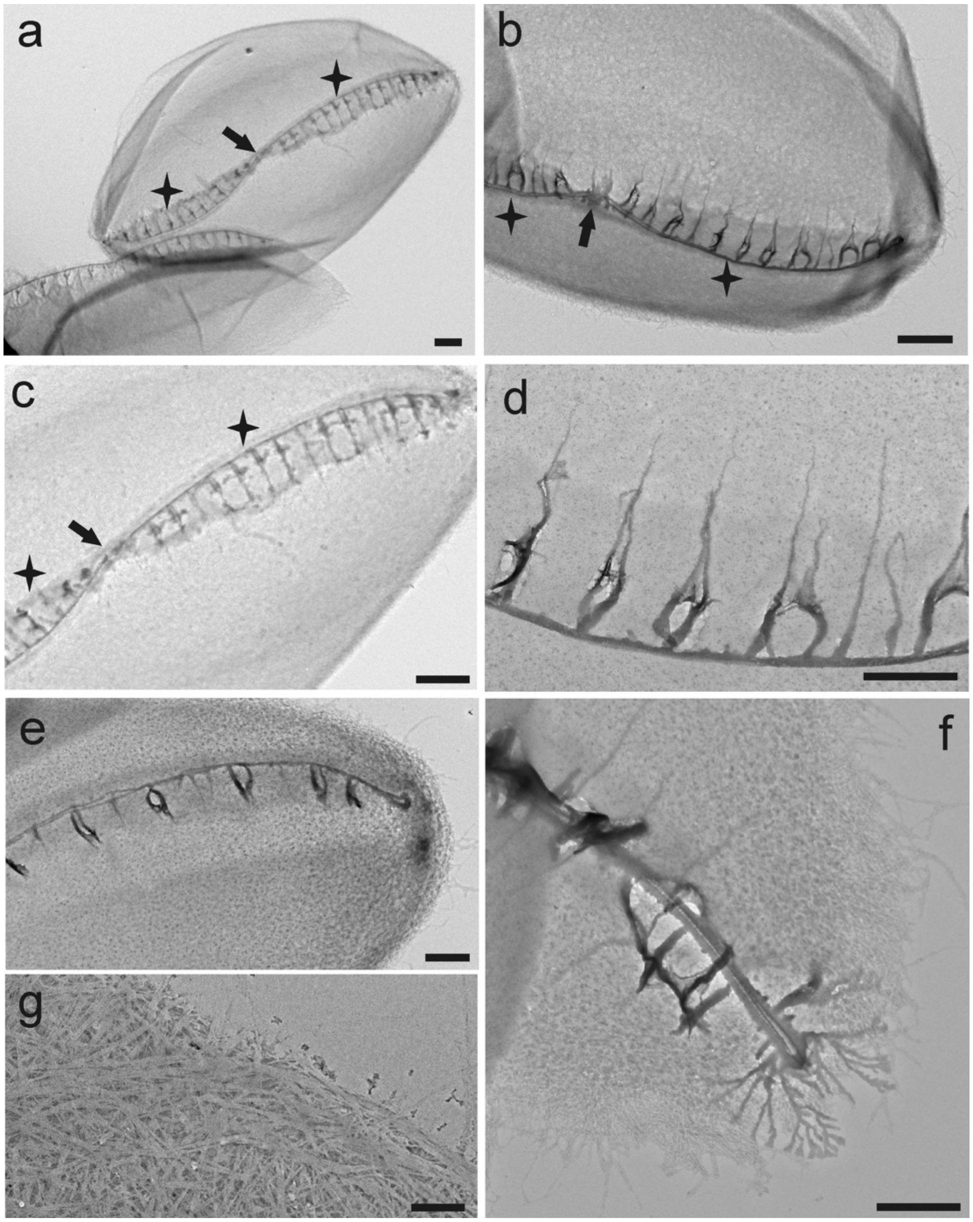

Supplementary Fig. 5. *Epithemia catenata* sp. nov. TEM micrographs.

a, **b**, **c**, **d**, valves with structureless walls and keeled fibulate raphe running from pole to pole. Stars are indicating both raphe branches separated by a central node (arrow); **a**, **c—**same valve; **b**, **d—**same valve. **d**, valve detail with fibulae. **e**, **f**, details of the distal raphe with branching structure (**f**). **g**, microfibrils are the only visible structural components of the valve walls. All images are from hydrogen peroxide-cleaned frustules. Scale bars are 2 µm (**a**, **b**), 1 µm (**c-f**) and 200 nm (**g**).

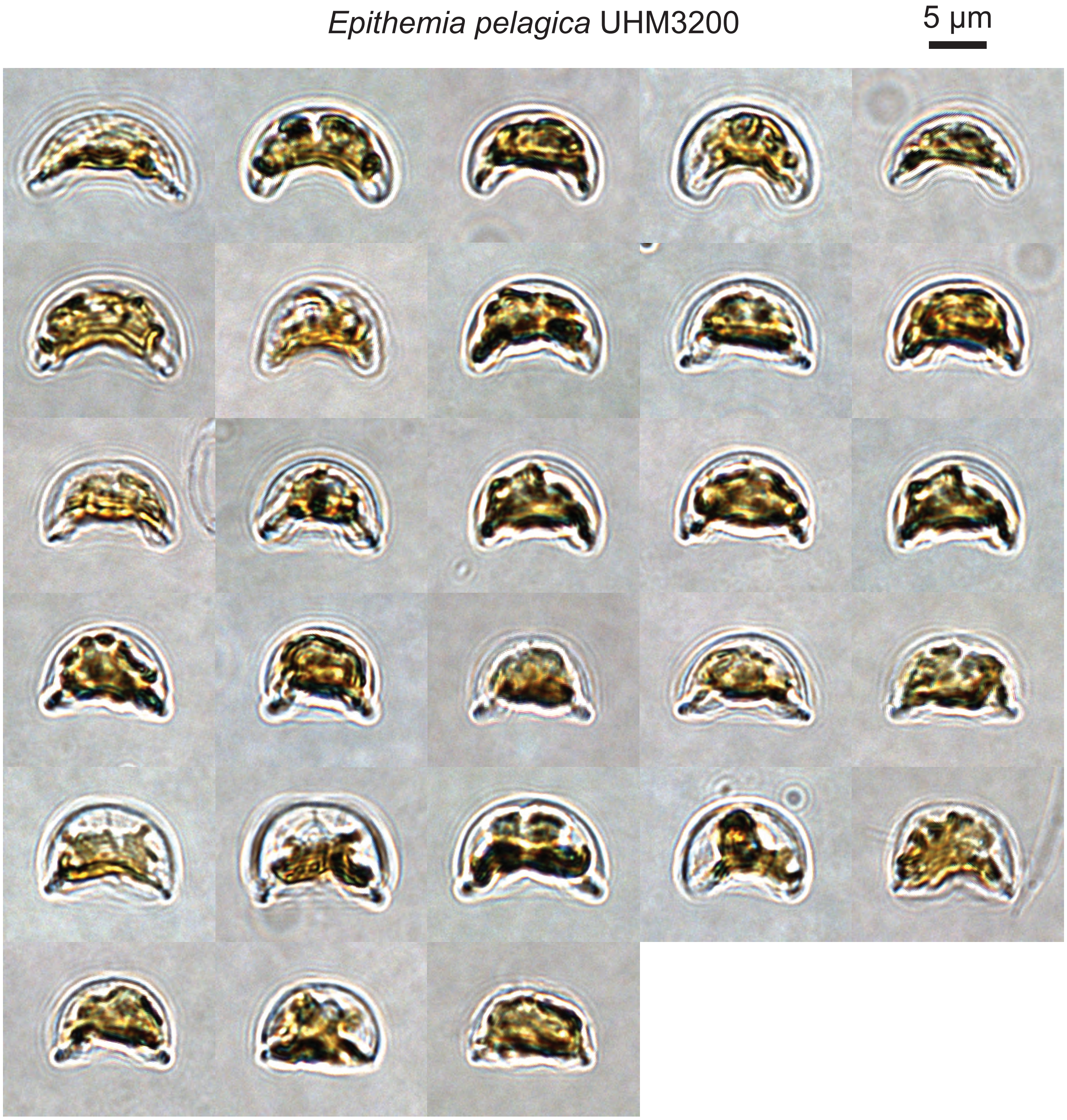

Supplementary Fig. 6. Light micrographs of live *E. pelagica* UHM3200 cells in valve view, imaged using a Nikon Eclipse 90i microscope at 60x magnification. A subset of the cells used for size measurements is included to illustrate possible morphological variability. Images were taken from two independent, exponentially growing cultures.

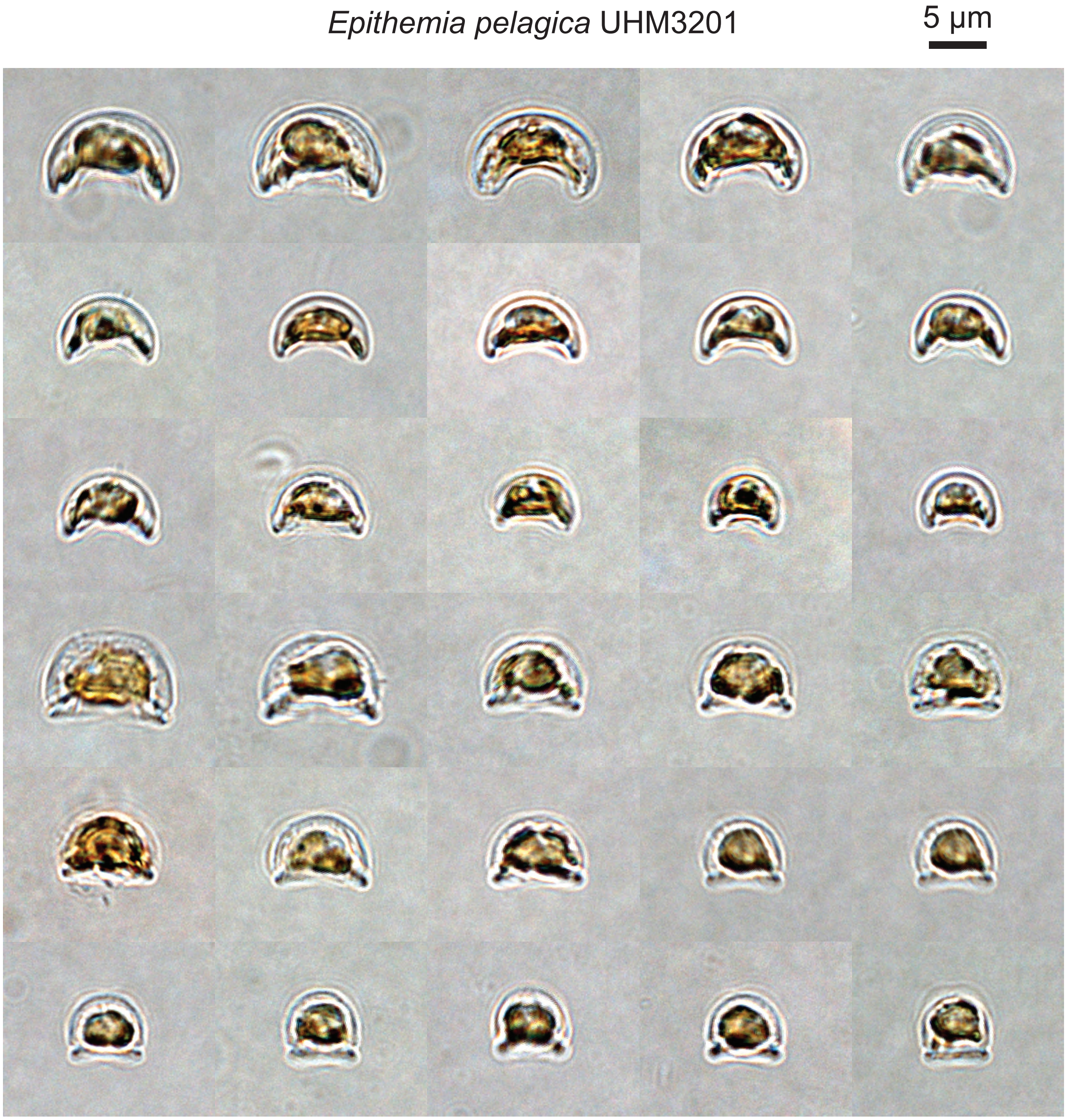

Supplementary Fig. 7. Light micrographs of live *E. pelagica* UHM3201 cells in valve view, imaged using a Nikon Eclipse 90i microscope at 60x magnification. A subset of the cells used for size measurements is included to illustrate possible morphological variability. Images were taken from two independent, exponentially growing cultures.

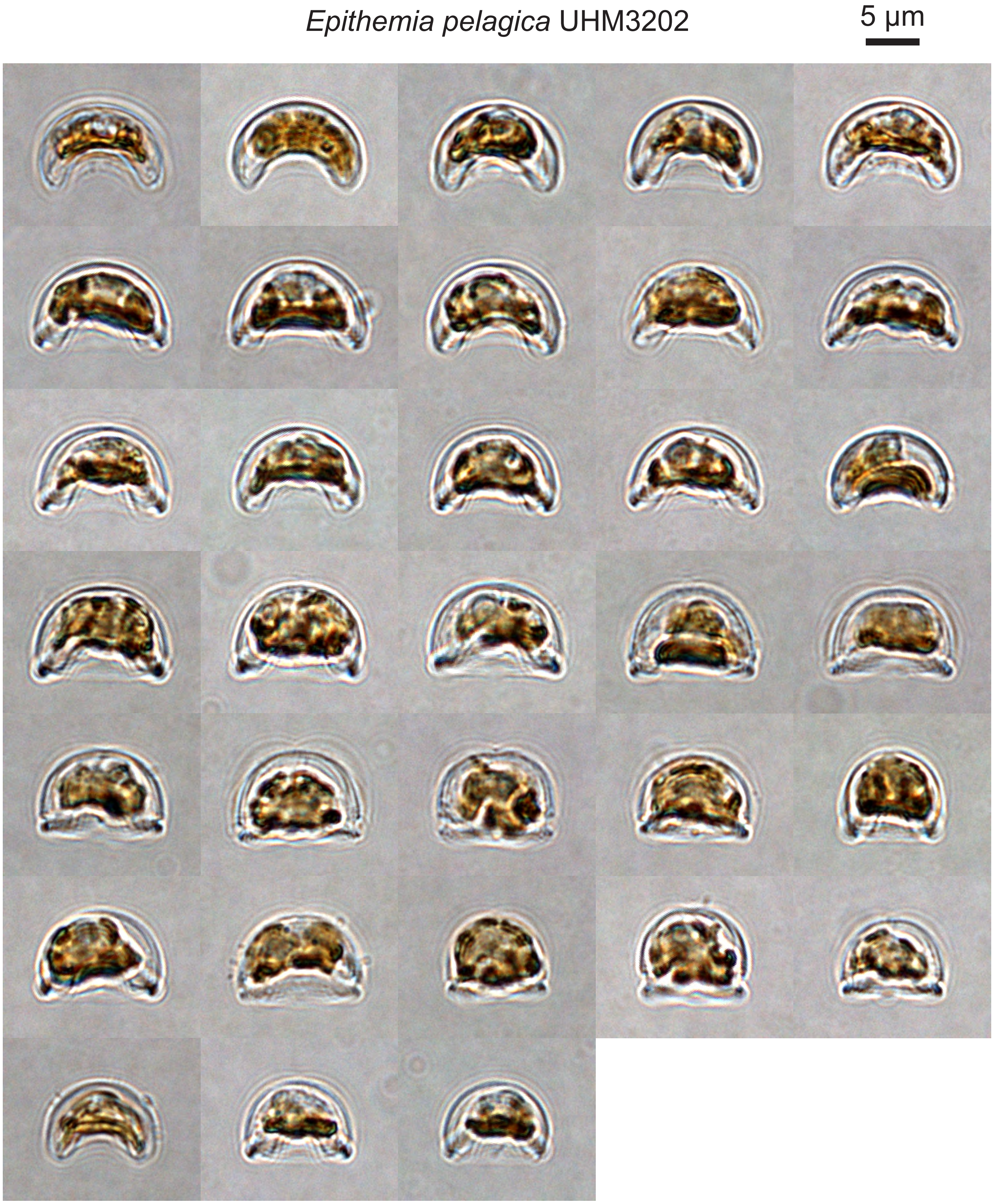

Supplementary Fig. 8. Light micrographs of live *E. pelagica* UHM3202 cells in valve view, imaged using a Nikon Eclipse 90i microscope at 60x magnification. A subset of the cells used for size measurements is included to illustrate possible morphological variability. Images were taken from two independent, exponentially growing cultures.

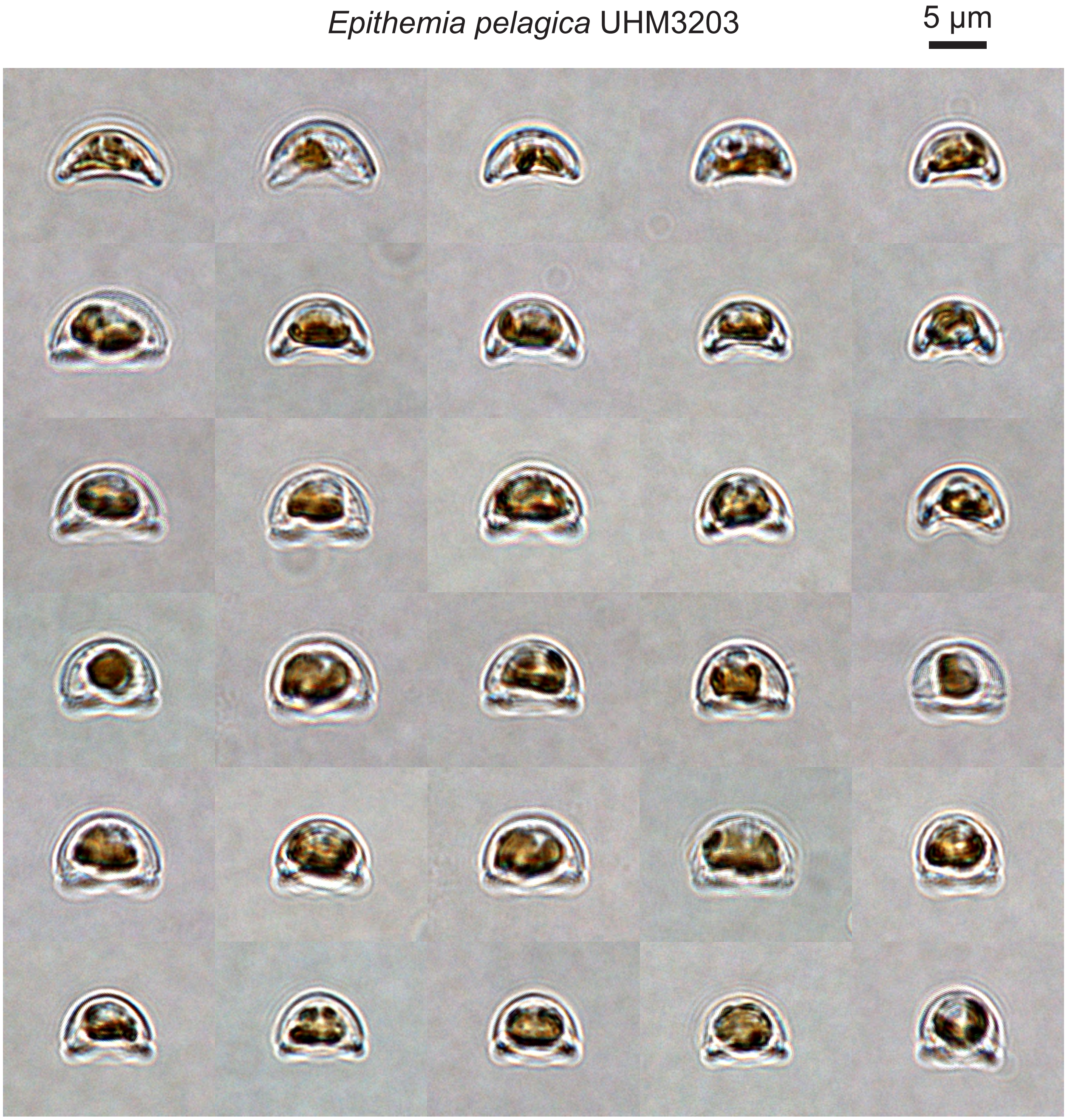

Supplementary Fig. 9. Light micrographs of live *E. pelagica* UHM3203 cells in valve view, imaged using a Nikon Eclipse 90i microscope at 60x magnification. A subset of the cells used for size measurements is included to illustrate possible morphological variability. Images were taken from two independent, exponentially growing cultures.

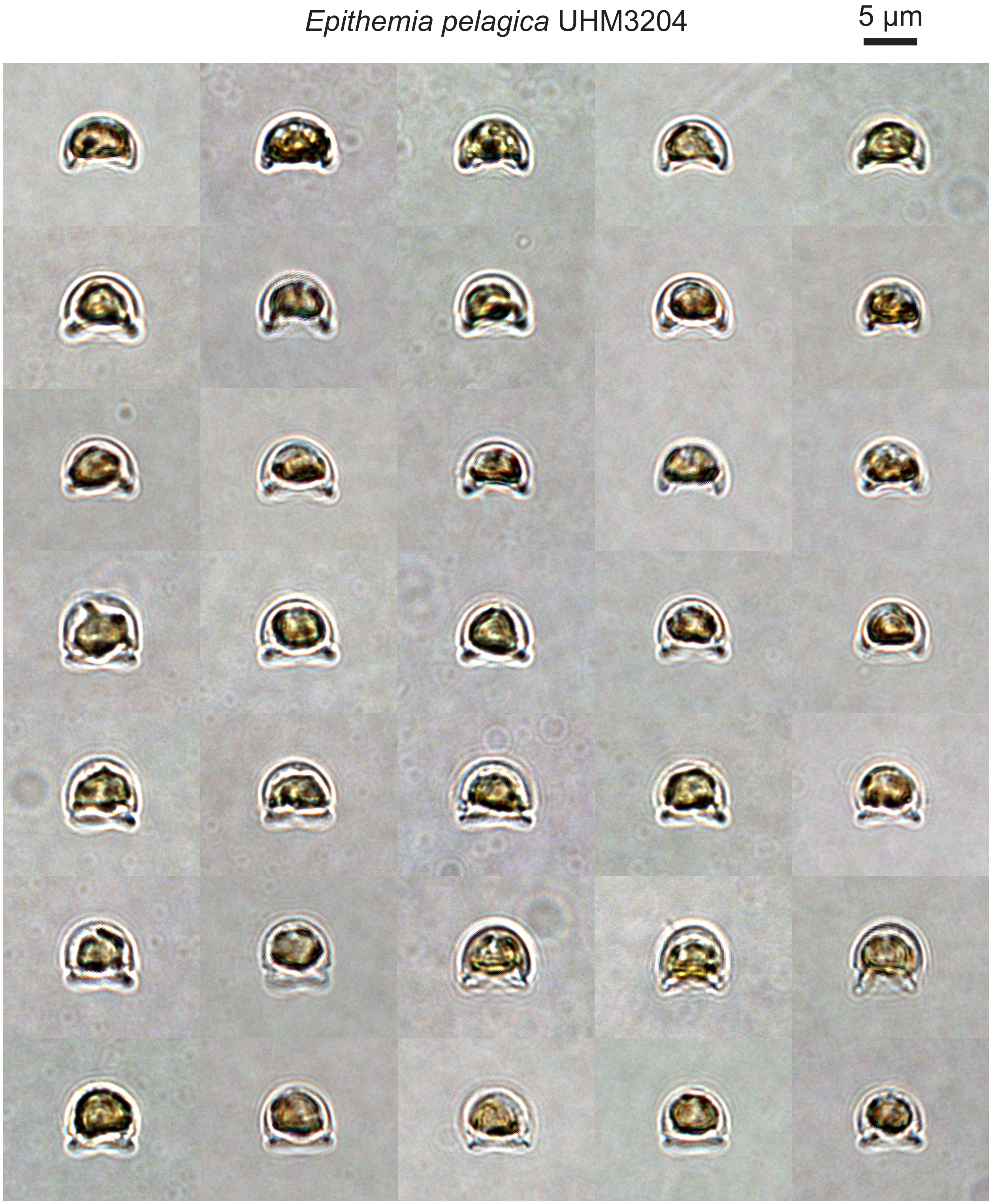

Supplementary Fig. 10. Light micrographs of live *E. pelagica* UHM3204 cells in valve view, imaged using a Nikon Eclipse 90i microscope at 60x magnification. A subset of the cells used for size measurements is included to illustrate possible morphological variability. Images were taken from two independent, exponentially growing cultures.

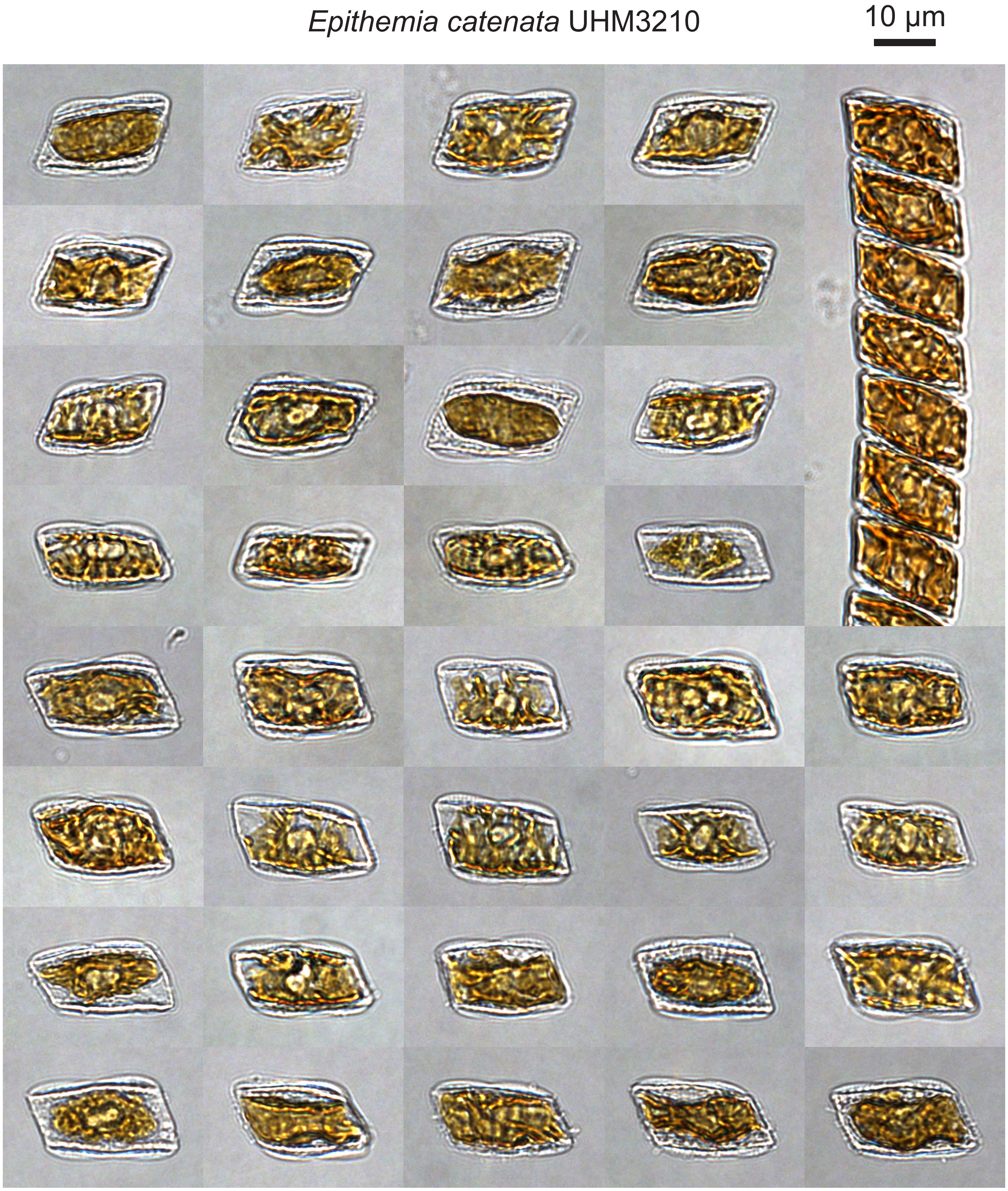

Supplementary Fig. 11. Light micrographs of live *E. catenata* UHM3210 cells in valve view, imaged using a Nikon Eclipse 90i microscope at 60x magnification. A subset of the cells used for size measurements is included to illustrate possible morphological variability. Images were taken from two independent, exponentially growing cultures.

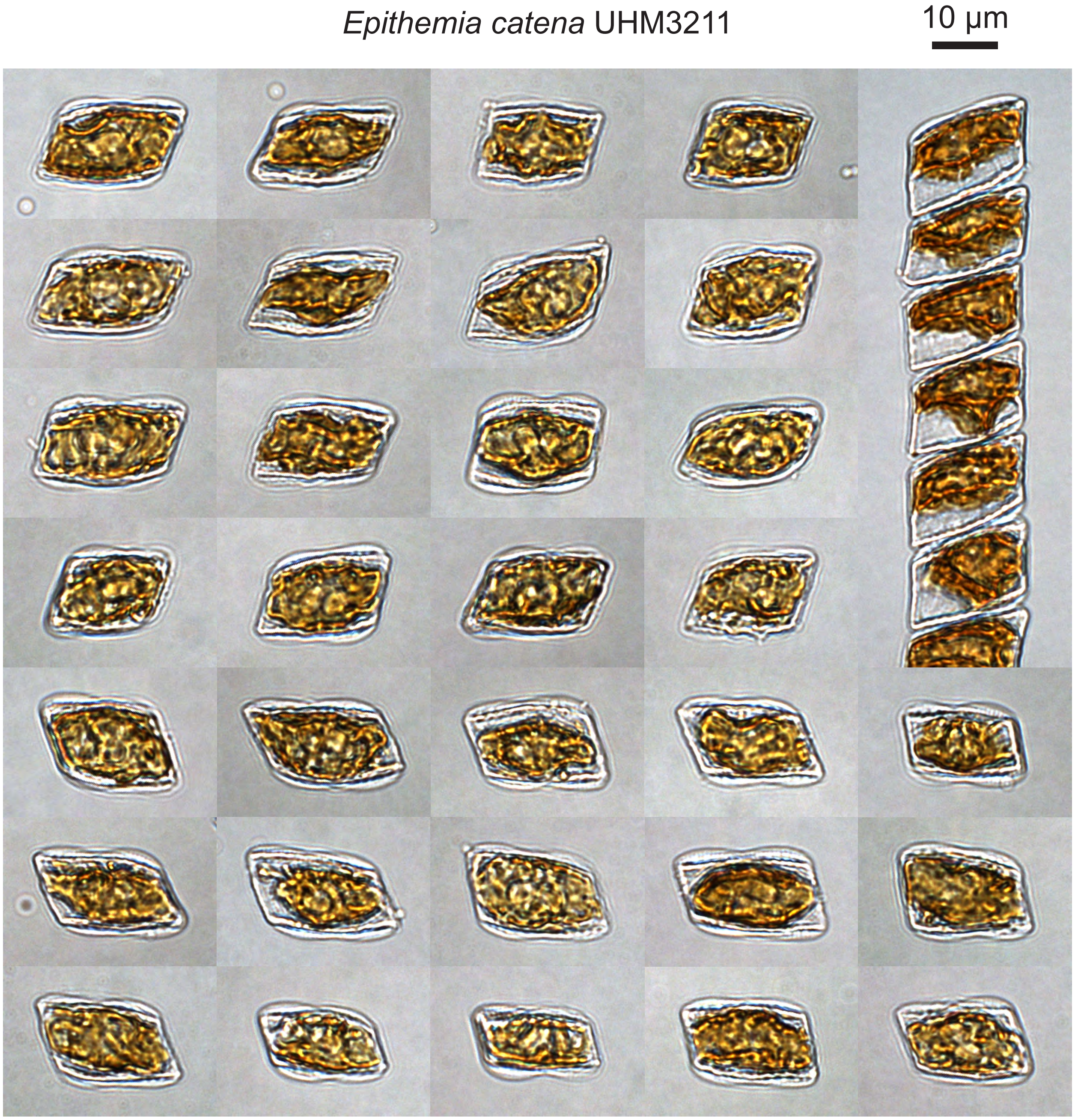

Supplementary Fig. 12. Light micrographs of live *E. catenata* UHM3211 cells in valve view, imaged using a Nikon Eclipse 90i microscope at 60x magnification. A subset of the cells used for size measurements is included to illustrate possible morphological variability. Images were taken from two independent, exponentially growing cultures.

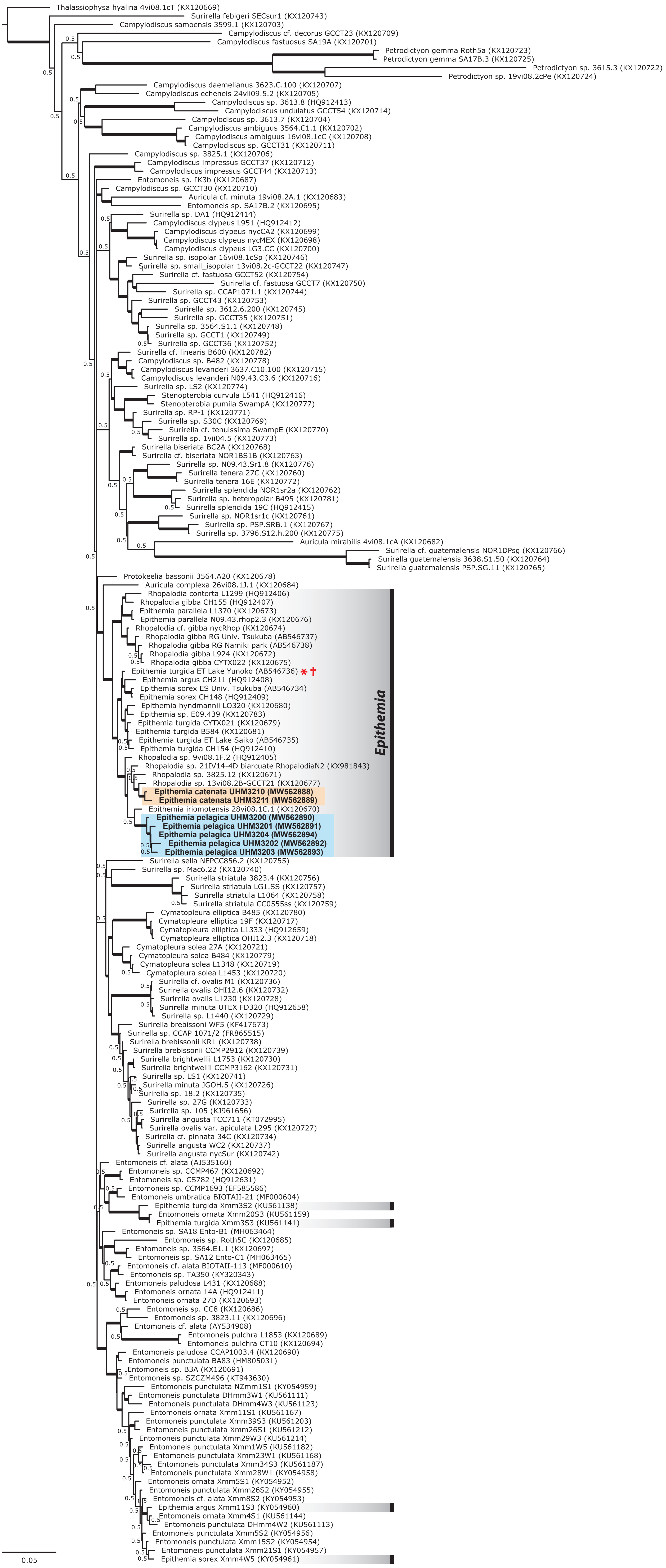

Supplementary Fig. 13. Bayesian majority consensus tree of Rhopalodiales and Surirellales diatoms, based on the small subunit ribosomal RNA gene (SSU/18S) aligned de novo using MAFFT (1,753 bp). Branches are labelled with posterior probability support values, and bold branches indicate complete support (posterior probability of 1). The top BLAST (megablast) hits in the NCBI non-redundant nucleotide database (nt, excluding environmental sequences) for *E. pelagica* and *E. catenata* are marked with a red asterisk (*) and dagger (†), respectively. Phylogeny scales are in units of nt substitutions per site. Accession numbers for all sequences are provided in the Source Data file.

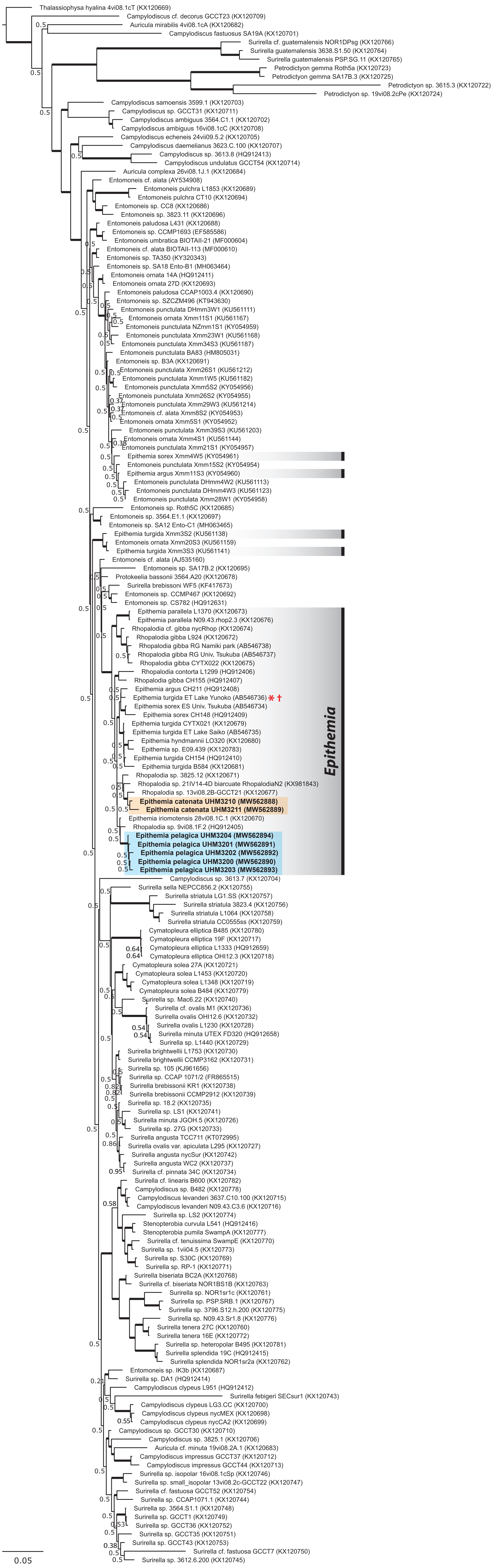

Supplementary Fig. 14. Bayesian majority consensus tree of Rhopalodiales and Surirellales diatoms, based on the small subunit ribosomal RNA gene (SSU/18S) aligned according to the global SILVA alignment for SSU genes using SINA (1589 bp). Branches are labelled with posterior probability support values, and bold branches indicate complete support (posterior probability of 1). The top BLAST (megablast) hits in the NCBI non-redundant nucleotide database (nt, excluding environmental sequences) for *E. pelagica* and *E. catenata* are marked with a red asterisk (*) and dagger (†), respectively. Phylogeny scale is in units of nt substitutions per site. Accession numbers for all sequences are provided in the Source Data file.

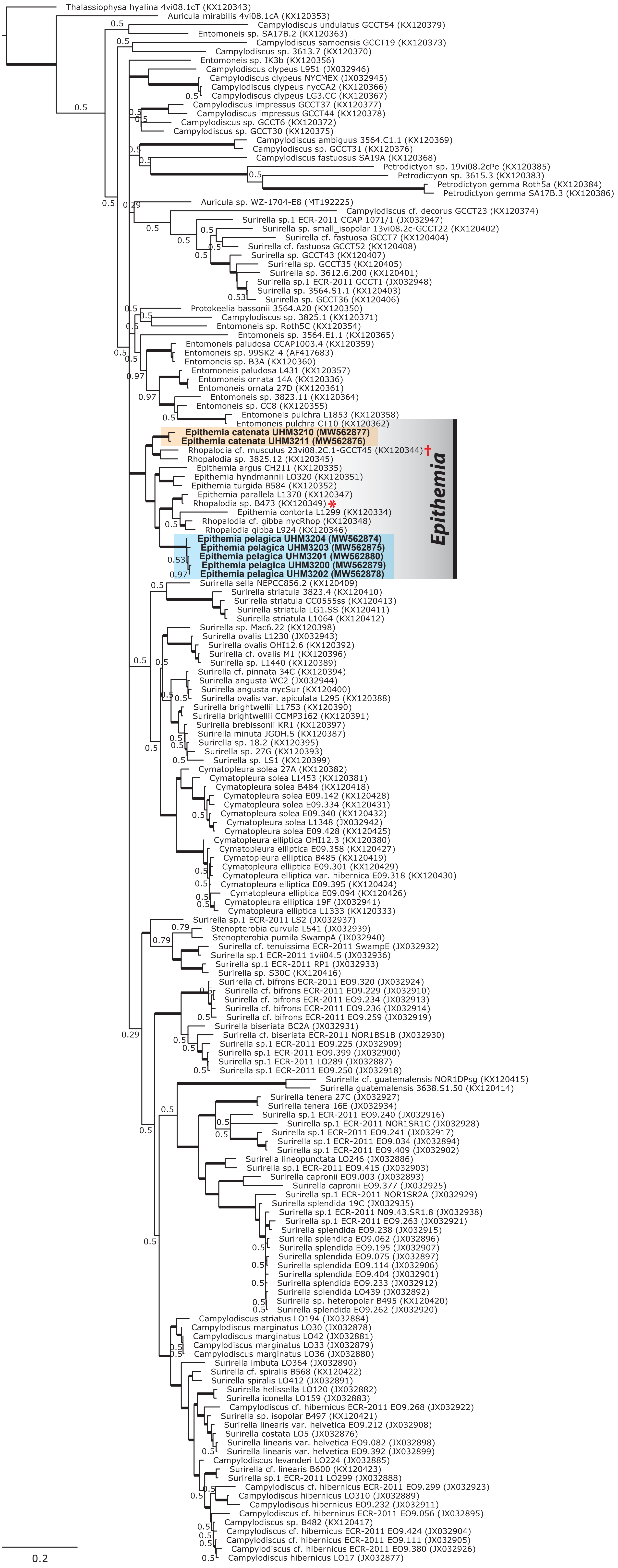

Supplementary Fig. 15. Bayesian majority consensus tree of Rhopalodiales and Surirellales diatoms, based on the large subunit ribosomal RNA gene (LSU/28S) aligned de novo using MAFFT (507 bp). Branches are labelled with posterior probability support values, and bold branches indicate complete support (posterior probability of 1). The top BLAST (megablast) hits in the NCBI non-redundant nucleotide database (nt, excluding environmental sequences) for *E. pelagica* and *E. catenata* are marked with a red asterisk (*) and dagger (†), respectively. Phylogeny scale is in units of nt substitutions per site. Accession numbers for all sequences are provided in the Source Data file.

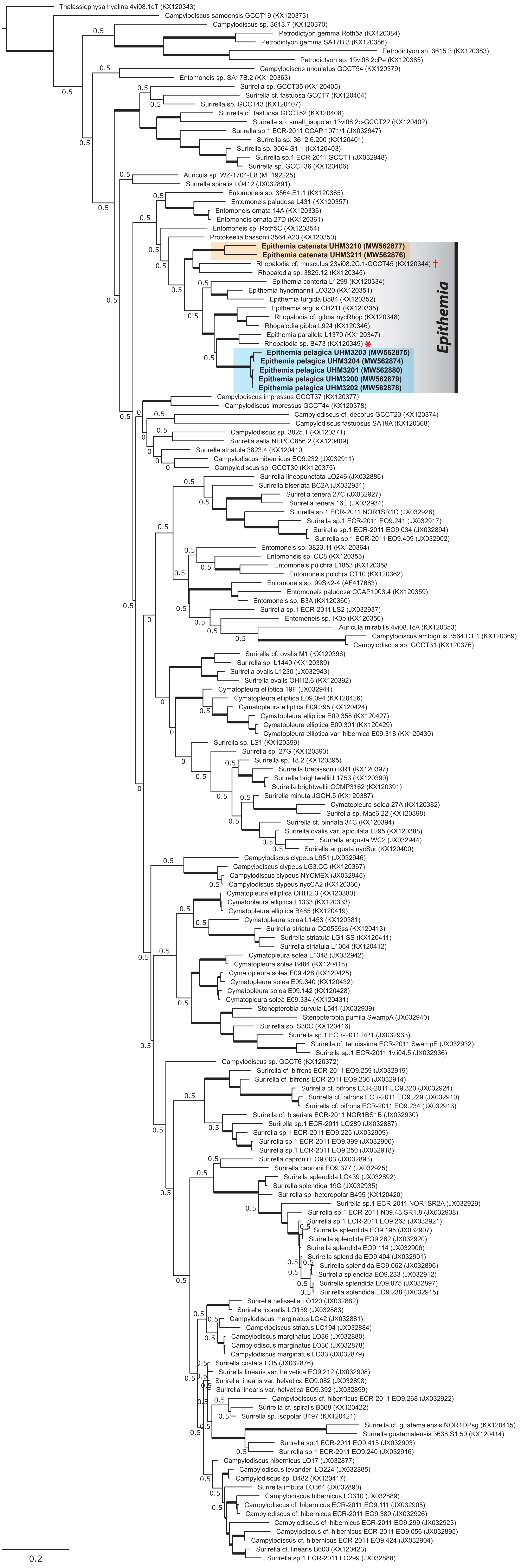

Supplementary Fig. 16. Bayesian majority consensus tree of Rhopalodiales and Surirellales diatoms, based on the large subunit ribosomal RNA gene (LSU/28S) aligned according to the global SILVA alignment for LSU genes using SINA (1105 bp). Branches are labelled with posterior probability support values, and bold branches indicate complete support (posterior probability of 1). The top BLAST (megablast) hits in the NCBI non-redundant nucleotide database (nt, excluding environmental sequences) for *E. pelagica* and *E. catenata* are marked with a red asterisk (*) and dagger (†), respectively. Phylogeny scale is in units of nt substitutions per site. Accession numbers for all sequences are provided in the Source Data file.

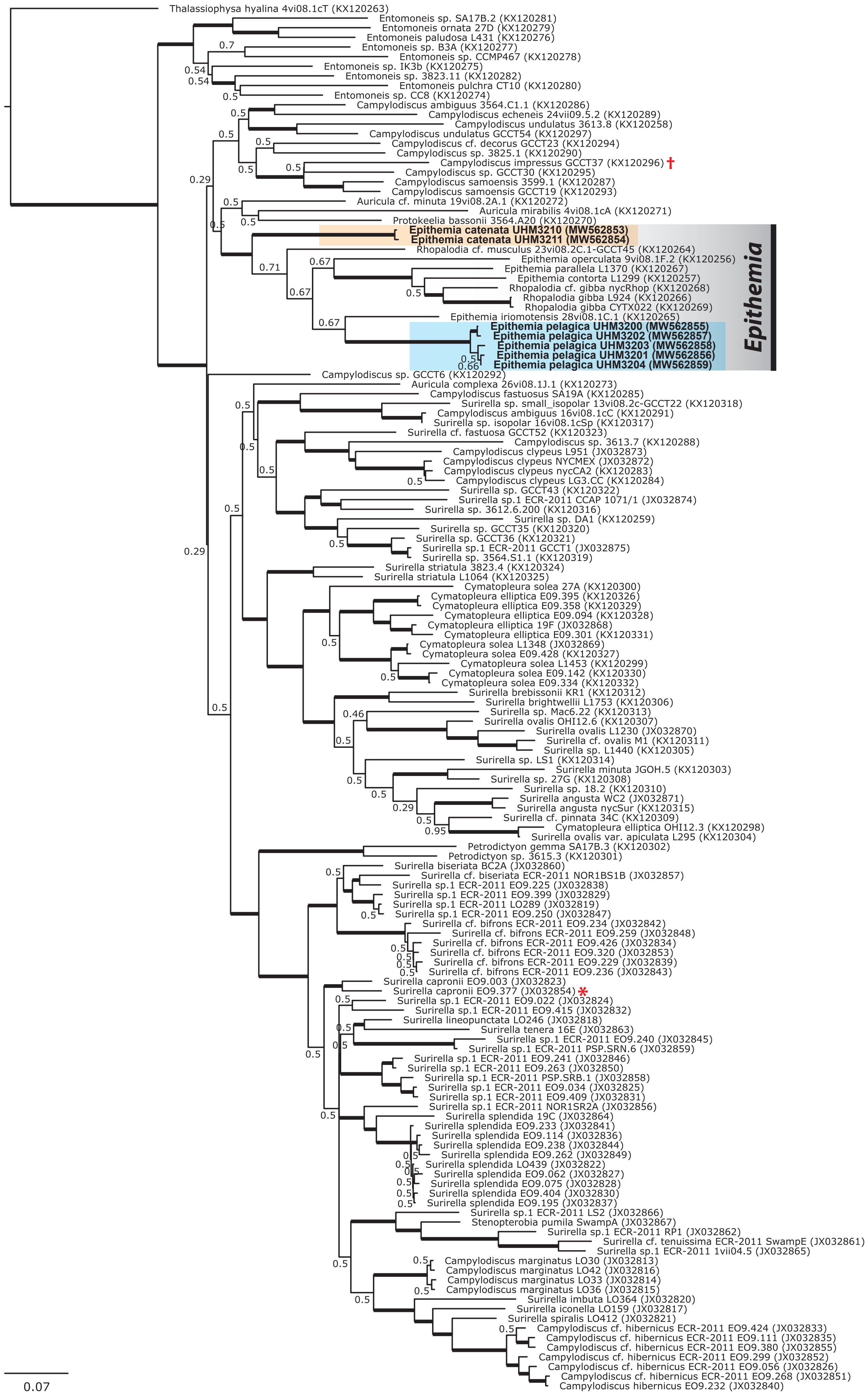

Supplementary Fig. 17. Bayesian majority consensus tree of Rhopalodiales and Surirellales diatoms, based on the apocytochrome b gene (*cob*; 529 bp). Branches are labelled with posterior probability support values, and bold branches indicate complete support (posterior probability of 1). The top BLAST (megablast) hits in the NCBI non-redundant nucleotide database (nt, excluding environmental sequences) for *E. pelagica* and *E. catenata* are marked with a red asterisk (*) and dagger (†), respectively. Phylogeny scale is in units of nt substitutions per site. Accession numbers for all sequences are provided in the Source Data file.

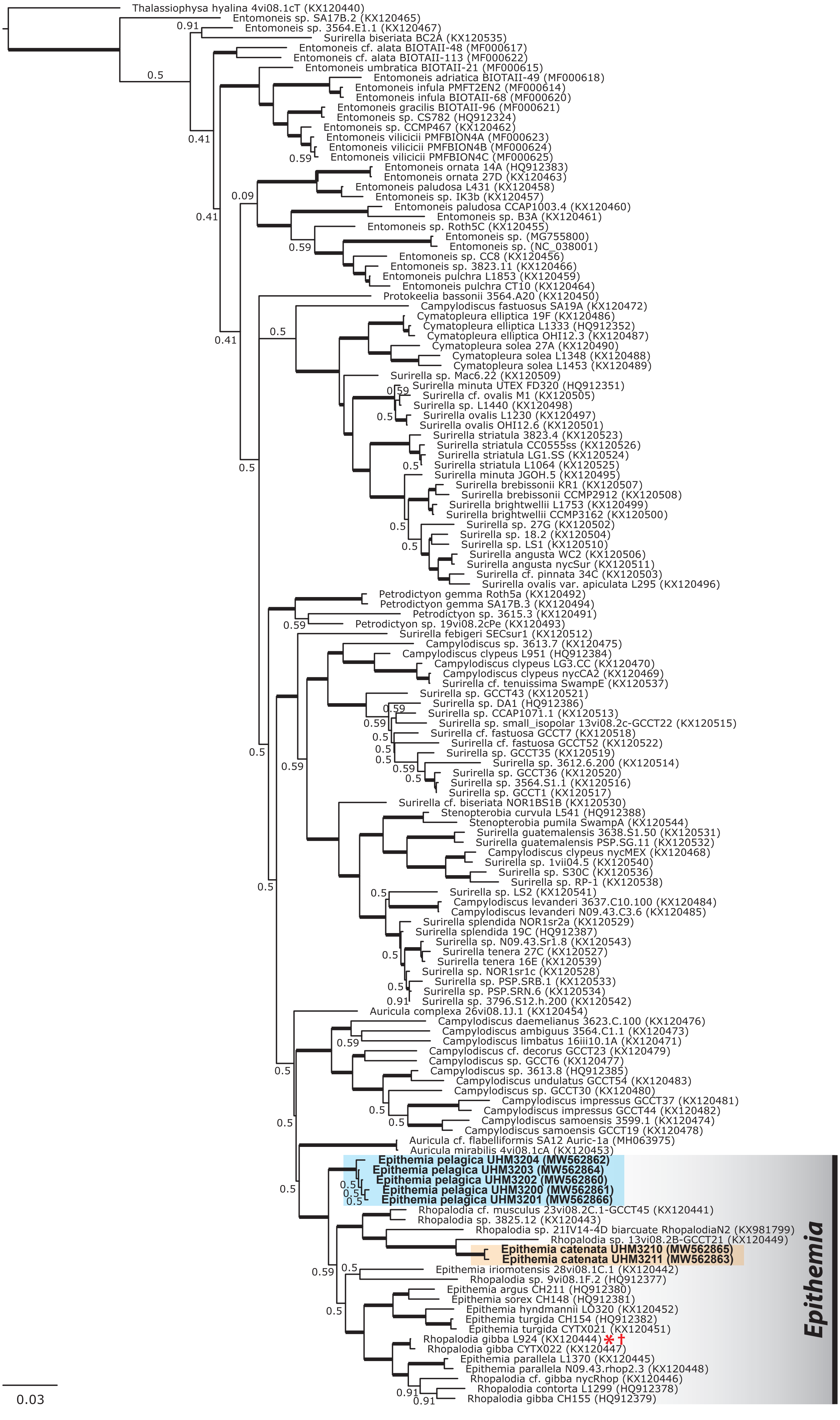

Supplementary Fig. 18. Bayesian majority consensus tree of Rhopalodiales and Surirellales diatoms, based on the photosystem II CP43 reaction center protein gene (*psbC*; 986 bp). Branches are labelled with posterior probability support values, and bold branches indicate complete support (posterior probability of 1). The top BLAST (megablast) hits in the NCBI non-redundant nucleotide database (nt, excluding environmental sequences) for *E. pelagica* and *E. catenata* are marked with a red asterisk (*) and dagger (†), respectively. Phylogeny scale is in units of nt substitutions per site. Accession numbers for all sequences are provided in the Source Data file.

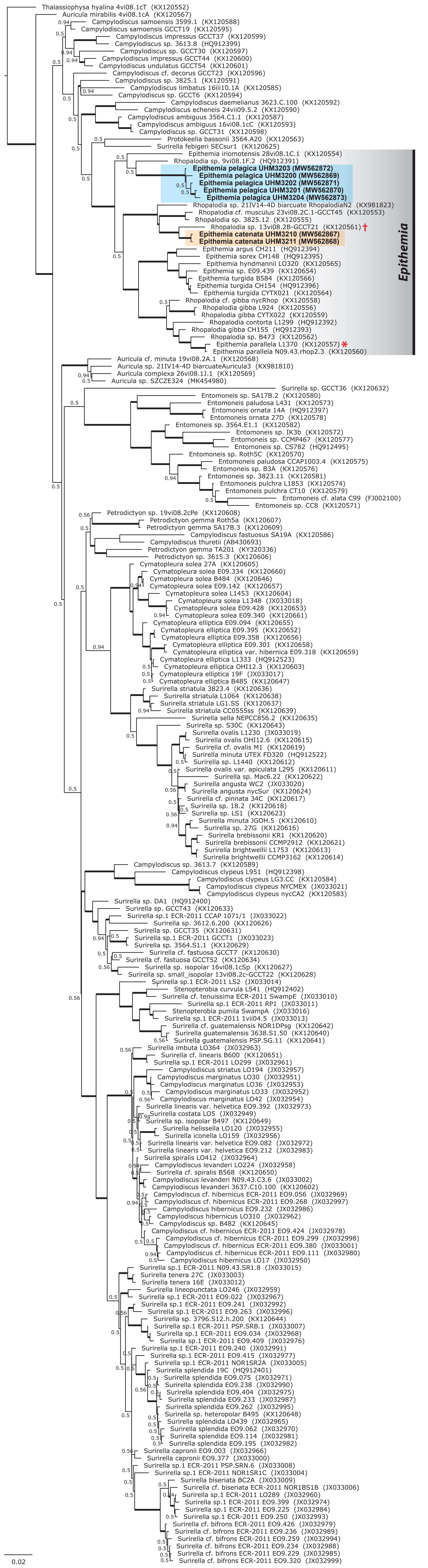

Supplementary Fig. 19. Bayesian majority consensus tree of Rhopalodiales and Surirellales diatoms, based on the ribulose-bisphosphate carboxylase gene (*rbcL*; 1,388 bp). Branches are labelled with posterior probability support values, and bold branches indicate complete support (posterior probability of 1). The top BLAST (megablast) hits in the NCBI non-redundant nucleotide database (nt, excluding environmental sequences) for *E. pelagica* and *E. catenata* are marked with a red asterisk (*) and dagger (†), respectively. Phylogeny scale is in units of nt substitutions per site. Accession numbers for all sequences are provided in the Source Data file.

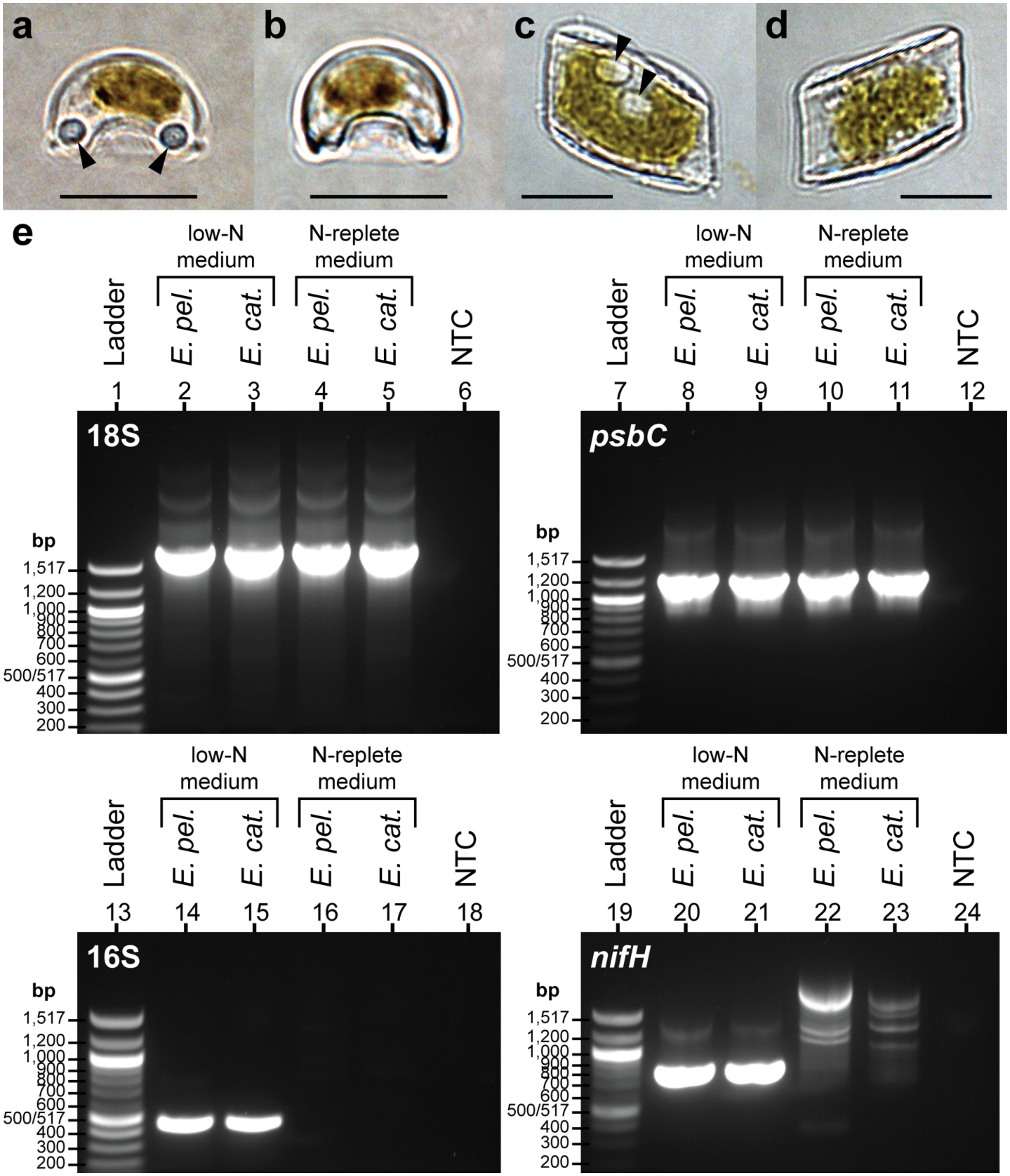

Supplementary Fig. 20. Light micrographs and PCR amplifications demonstrating the loss of endosymbionts from *Epithemia* cultures UHM3200 and UHM3210 after extended propagation in nitrogen-replete medium (K medium). **a–d**, light micrographs of *E. pelagica* (**a, b**) and *E. catenata* (**c, d**), grown in either low-nitrogen medium (**a, c**) or nitrogen-replete K medium (**b, d**). Cell samples were osmotically shocked with ultrapure water to improve visualization of the endosymbionts. All scale bars are 10 µm and black arrows indicate the presence of

endosymbionts. The long-term growth experiments represented here, which investigate the loss of endosymbionts from cells grown in nitrogen-replete medium, were performed once for each *E. pelagica* and *E. catenata*. **e**, gel electrophoretic analysis of PCR amplifications performed on *E. pelagica* DNA extracts (lanes 2, 4, 8, 10, 14, 16, 20, 22) and *E. catenata* DNA extracts (lanes 3, 5, 9, 11, 15, 17, 21, 23) from cultures grown in either low-nitrogen medium (lanes 2, 3, 8, 9, 14, 15, 20, 21) or nitrogen-replete medium (lanes 4, 5, 10, 11, 16, 17, 22, 23). Diatom host marker genes 18S rRNA (lanes 2–6; 1,771 bp amplicon) and *psbC* (lanes 8–12; 1,252 bp amplicon) were successfully amplified from all culture DNA extracts, while the endosymbiont marker genes 16S rRNA (lanes 14–18; 458 bp amplicon) and *nifH* (lanes 20–24; 802 bp amplicon) were only successfully amplified from cultures grown in low-nitrogen medium. The *psbC* primer set is diatom-specific, while the 16S rRNA and *nifH* primer sets specifically target relatives of unicellular cyanobacterial diazotrophs (including *Epithemia* SBs). Note that in the case of *nifH*, the absence of appropriate template DNA led to increased non-specific amplification (lanes 22 and 23) but no amplification of the target genes. DNA ladders are provided in lanes 1, 7, 13, and 19, and the results of no template control (NTC) reactions for each gene are shown in lanes 6, 12, 18, and 24. For each gene, PCR reactions were prepared with 10 ng of template DNA (except for NTCs) using the same master mixes, and reactions were amplified for 35 cycles. Uncropped gel images are provided in the Source Data file.

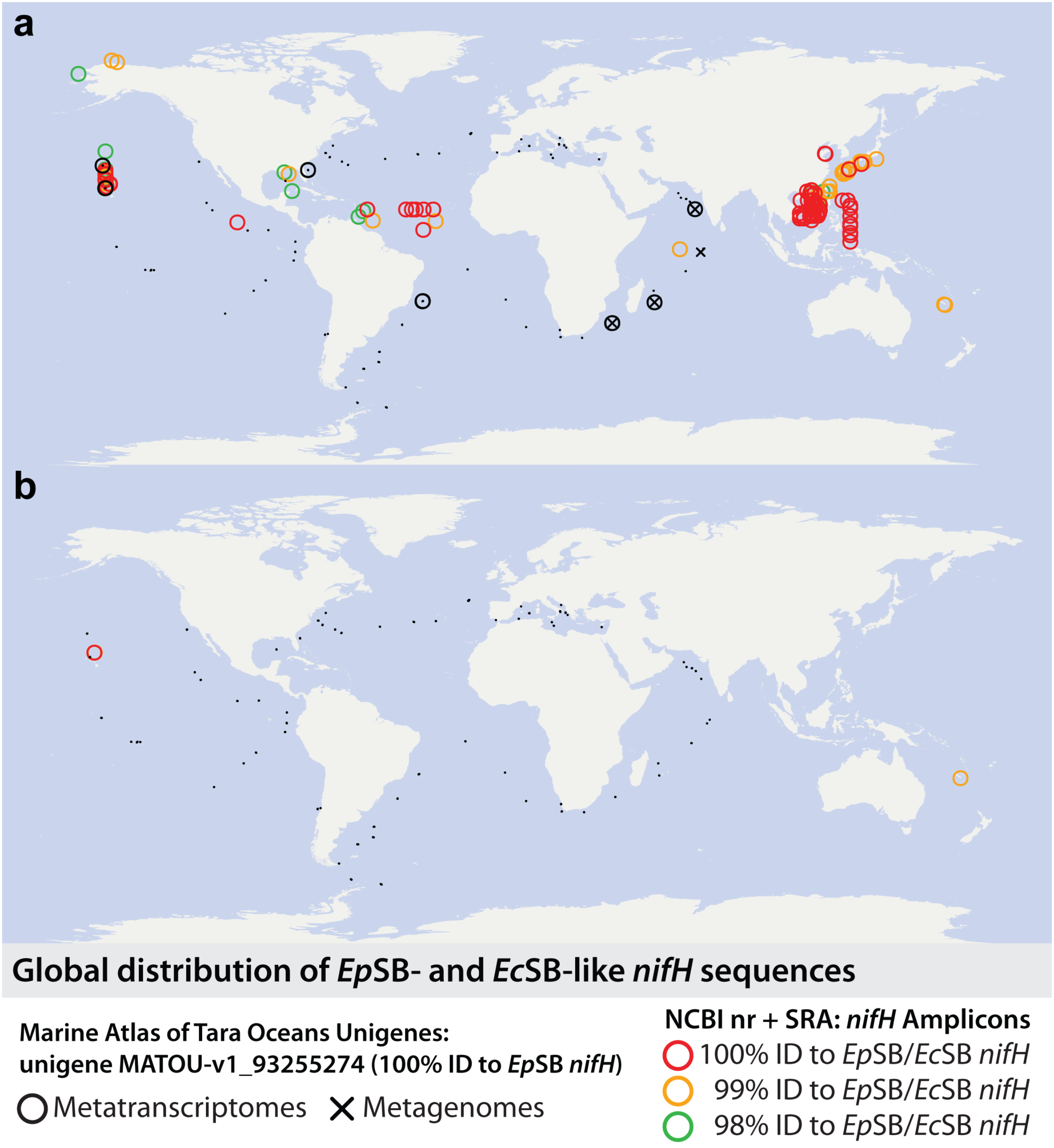

Supplementary Fig. 21. Global distribution of environmental sequences that share 98–100% nucleotide sequence identity with *nifH* phylotypes for either *E. pelagica* SB (**a**) or *E. catenata* SB (**b**). Datasets screened include the Marine Atlas of *Tara* Oceans Unigenes[1] and their representation in *Tara* Oceans metagenomes and metatranscriptomes, as well as *nifH* amplicons published in NCBI's non-redundant (nt) nucleotide[2] and Sequence Read Archive (SRA)[3] databases. Black dots indicate the location of *Tara* Oceans stations analyzed in MATOU. Source data are provided in the Source Data file.

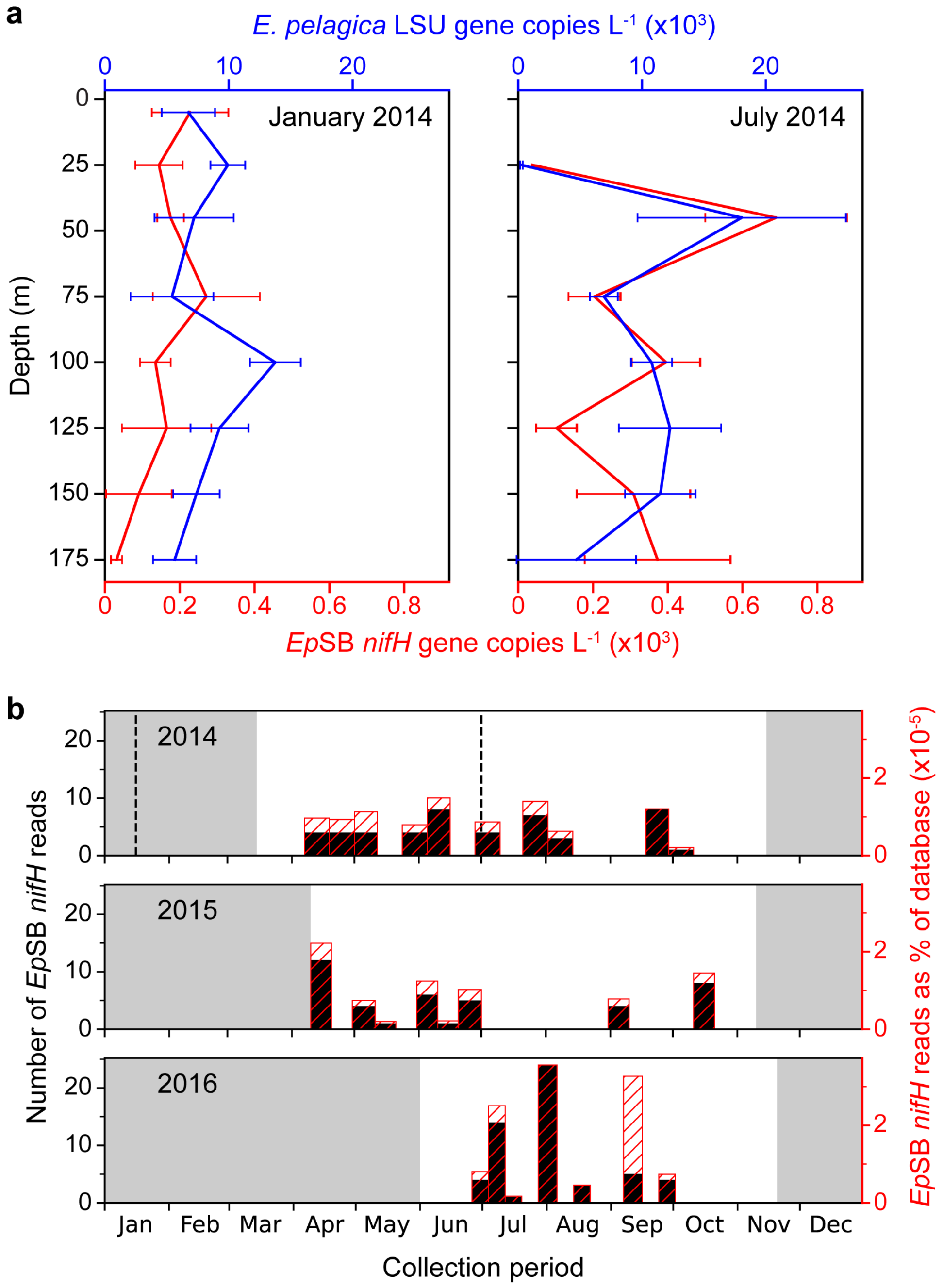

Supplementary Fig. 22. Observations of *E. pelagica* and *Ep*SB in samples collected at Station ALOHA. **a**, quantitative PCR (qPCR) measurements of gene copy concentrations for *E. pelagica* (LSU; blue) and *Ep*SB (*nifH*; red) in euphotic zone samples collected in January and July of 2014. Plotted are the mean values and standard deviation (error bars) of n=4 replicate qPCR reactions for each sample and gene. The highest observed concentrations for both *nifH* and LSU gene copies coincided in July at 45m. However, smaller peaks in LSU concentrations were observed at deeper depths without coinciding peaks in *nifH*. **b**, *Ep*SB *nifH* sequences (100% nucleotide sequence identity) in metagenomes prepared from sediment trap material collected at 4000 m depth at Station ALOHA[4,5]. Values are presented as either a read count (black) or as a percentage of total reads in the associated metagenome (red). Bar widths correspond to the

dates encompassed by each trap sample. Shaded regions of the plots represent unsampled time periods, and the vertical dashed lines in 2014 represent the collection dates for the qPCR samples shown in **a**. Source data are provided in the Source Data file.

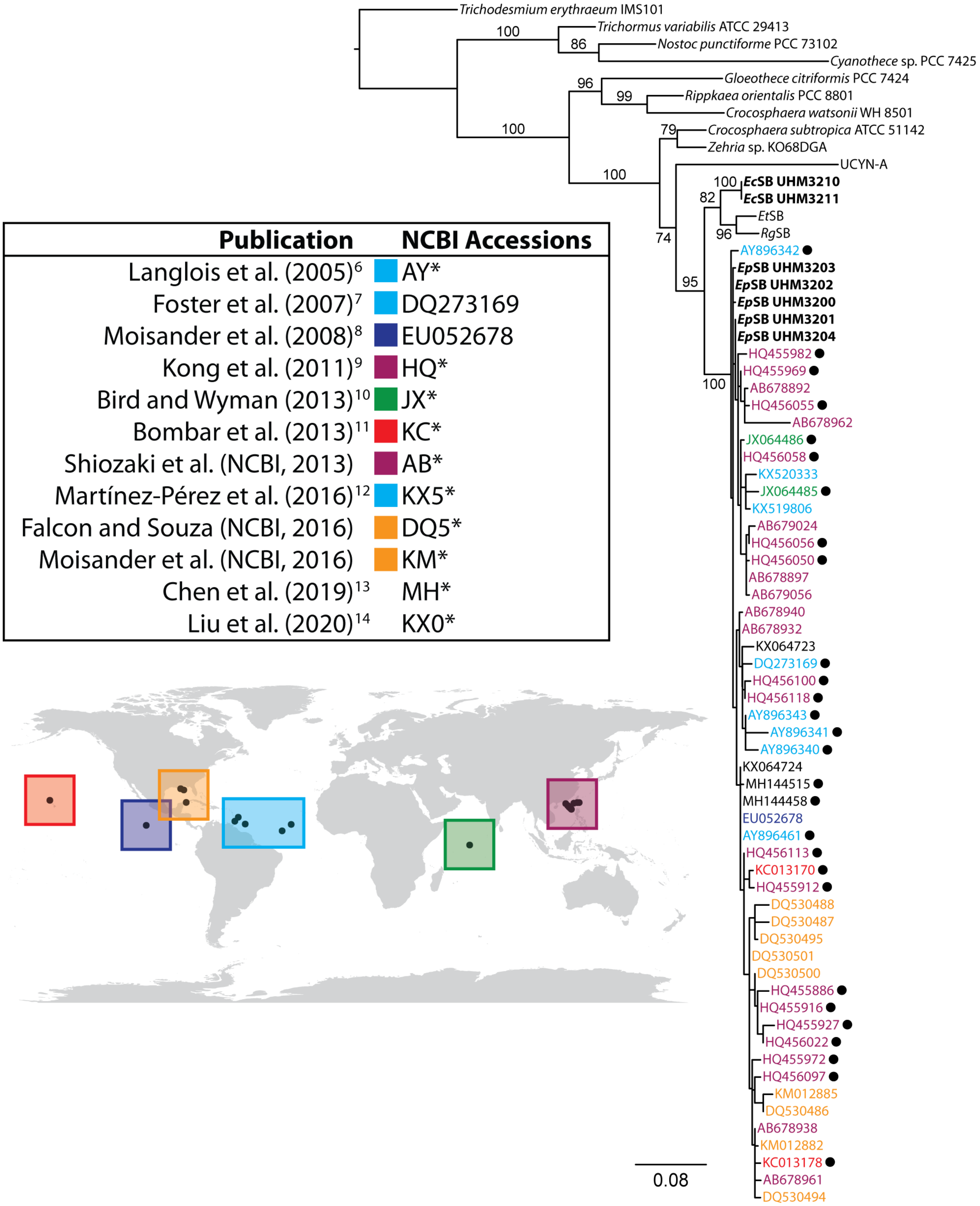

Supplementary Fig. 23. Maximum likelihood phylogeny illustrating the prior detection of *Epithemia* SB-like *nifH* sequences in environmental samples. The tree includes all environmental *nifH* sequences (>300 bp) in the NCBI non-redundant nucleotide (nt) database that share >95% nucleotide identity with *Ep*SB and *Ec*SB. Environmental sequences are labelled with their accession number, and their associated publication is provided in the table

(asterisks in the table accession numbers represent wildcards). Sequences previously identified as either “UCYN-C”, “*Cyanothece*-like”, or “unicellular cyanobacteria” are marked by filled circles; the remaining environmental sequences are either unpublished or were not identified in the associated study. Label colors correspond to the geographic origin of the samples, as highlighted on the map (note: the size of the highlighted boxes is arbitrary). The coordinates for these samples are plotted, except for KX064723 and KX064724 (from the South China Sea) and MH144515 and MH144458 (from the western North Pacific Ocean), where this information was unavailable. Bootstrap support values (% of 1000 replicates) are provided for major branches, and the scale bar is in units of nucleotide substitutions per site. Accession numbers for all sequences are provided in the Source Data file.

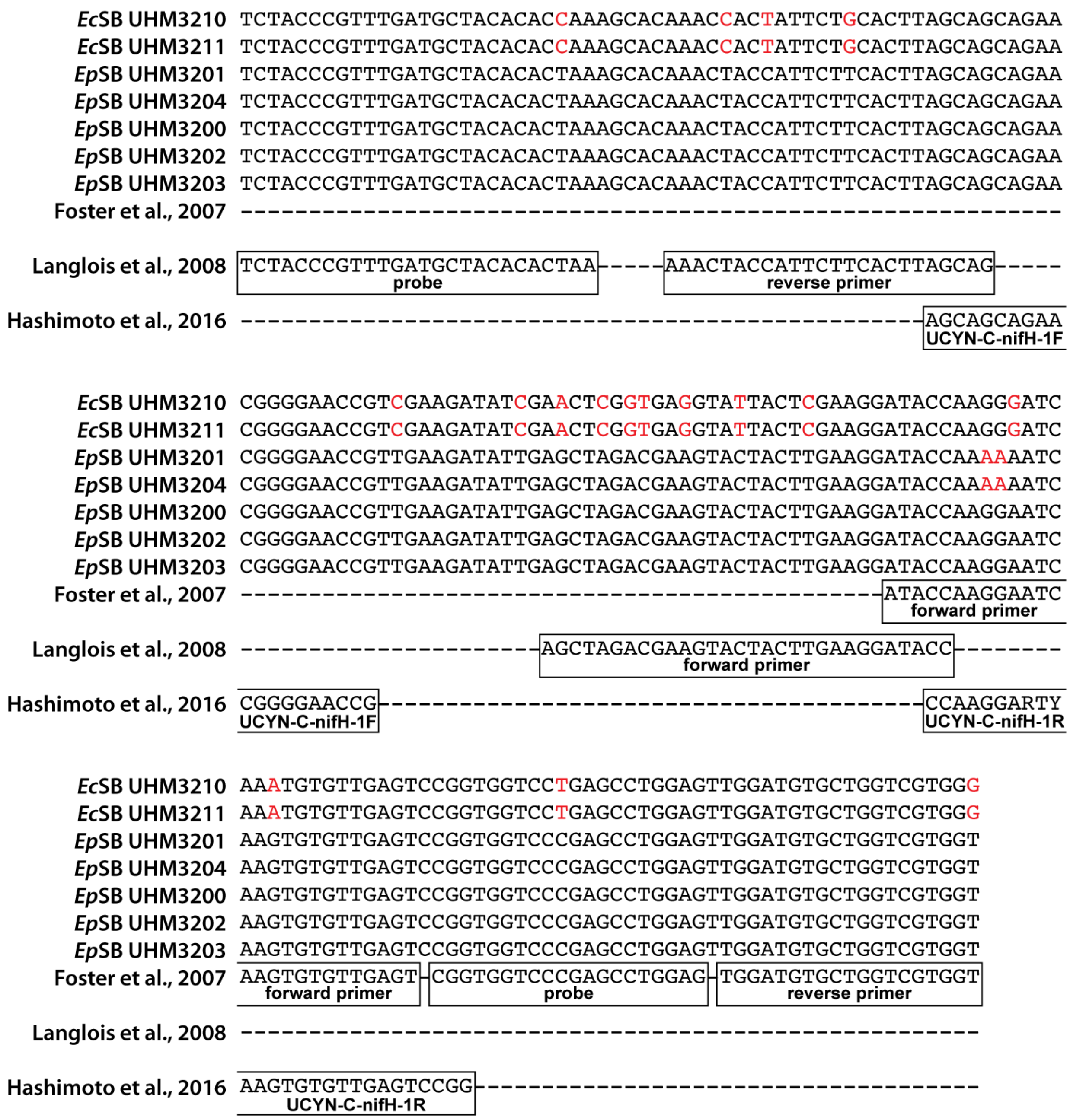

Supplementary Fig. 24. Alignment of *Ec*SB and *Ep*SB *nifH* sequences with previously published primers and probes targeting the UCYN-C clade[7,15,16]. Bases in red represent disagreements with both the consensus sequence and primer/probe sequences. The primer and probe sequences displayed here have been reoriented for the alignment and thus may represent reverse complements of published sequences.

Supplementary Tables

Strain Metadata					NCBI Accession Numbers						
Species	Strain	Collection Date	Collection Depth (m)	HOT Cruise Number	18S rRNA	LSU rRNA	*rbcL*	*psbC*	*Cob*	16S rRNA	*nifH*
E. pelagica	UHM3200	2014-10-15	25	266	MW562890	MW562879	MW562869	MW562861	MW562855	MW562883	MW562848
E. pelagica	UHM3201	2019-02-21	25	310	MW562891	MW562880	MW562870	MW562866	MW562856	MW562884	MW562849
E. pelagica	UHM3202	2019-05-04	100	311	MW562892	MW562878	MW562871	MW562860	MW562857	MW562885	MW562850
E. pelagica	UHM3203	2019-06-13	75	312	MW562893	MW562875	MW562872	MW562864	MW562858	MW562886	MW562851
E. pelagica	UHM3204	2019-07-03	100	313	MW562894	MW562874	MW562873	MW562862	MW562859	MW562887	MW562852
E. catenata	UHM3210	2019-02-21	25	310	MW562888	MW562877	MW562867	MW562865	MW562853	MW562881	MW562846
E. catenata	UHM3211	2019-05-04	75	311	MW562889	MW562876	MW562868	MW562863	MW562854	MW562882	MW562847

Supplementary Table 1. Metadata and NCBI accession numbers for all *Epithemia pelagica* and *Epithemia catenata* isolates.

Species	Strain	Micrograph Type & Cell Preparation Method							
		Light Micrographs: Live Cells	Light Micrographs: Osmotically Shocked Cells	Light Micrographs: H2O2-Cleaned Cells Mounted in Naphrax	Epifluorescence Micrographs: Autofluorescence of Released Endosymbionts	Epifluorescence Micrographs: SYBR Gold-Stained Cells	SEM Micrographs: Uncleaned Cells	SEM Micrographs: H2O2-Cleaned Cells	TEM Micrographs: H2O2-Cleaned Cells
E. pelagica	UHM3200	Fig 1b, Suppl. Fig. 6	Fig. 1c, Suppl. Fig. 18a,b		Fig. 1h–j	Fig. 1n	Suppl. Fig. 1i–k	Suppl. Figs. 1l–m, 2	
E. pelagica	UHM3201	Suppl. Fig. 7		Suppl. Fig. 1a–h					
E. pelagica	UHM3202	Suppl. Fig. 8							
E. pelagica	UHM3203	Suppl. Fig. 9							
E. pelagica	UHM3204	Suppl. Fig. 10							
E. catenata	UHM3210	Fig 1e, Suppl. Fig. 11	Fig. 1f, Suppl. Fig. 18c,d	Suppl. Fig. 3	Fig. 1k–m	Fig. 1o	Suppl. Fig. 4a–g	Suppl. Fig. 4h–k	Suppl. Fig. 5
E. catenata	UHM3211	Suppl. Fig. 12							

Supplementary Table 2. Key of *Epithemia* strains used in each set of micrograph images.

	Gene						
	SSU		LSU				
Phylogenetic Constraint	MAFFT	SINA	MAFFT	SINA	***cob***	***psbC***	***rbcL***
**1.** (*Thalassiophysa*, (*Protokeelia*, (*E. catenata* + All E/R)))	0.684/0.414	0.837/0.625	0.697/0.464	0.278/0.115	1/0.612	0.744/0.000757	0.807/0.555
**2.** (*Thalassiophysa*, (*Protokeelia*, ((*E. catenata* + Subset E/R), (Remaining E/R))))	0.0535/0.0256	0.442/0.17	0.8/0.528	1/0.867	0.816/0.414	1/0.928	1/0.569
**3.** (*Thalassiophysa*, ((*E. catenata* + Subset E/R), (*Protokeelia*, (Remaining E/R))))	0.037/0.00686	0.344/0.106	0.824/0.394	0.172/0.114	0.469/0.132	0.327/0.107	0.538/0.196
**4.** (*Thalassiophysa*, (*Protokeelia*, (*E. catenata*, (All E/R))))	0.0169/0.00374	0.107/0.0292	0.684/0.322	0.604/0.356	0.878/0.538	0.0005/2.51E-06	0.0658/0.0168
**5.** (*Thalassiophysa*, (*E. catenata*, (*Protokeelia*, (All E/R))))	0.0075/0.0047	0.153/0.0185	0.72/0.406	0.134/0.164	0.478/0.0803	0/1.81E-55	0.012/3.72E-05

Supplementary Table 3. Results of hypothesis testing for constrained diatom tree topologies. For each gene sequence alignment, p-values are shown for SH/AU tests[17,18]. The SSU and LSU genes were tested using sequences aligned either de novo using MAFFT[19] or according to the global SILVA alignments for SSU and LSU genes using SINA[20]. P-values in red indicate rejection of the tree topology ($p < 0.05$). "All E/R" stands for all *Epithemia*/*Rhopalodia* strains, except for *E. catenata*, "Subset E/R" stands for any of the four *Epithemia*/*Rhopalodia* strains closely related to *E. catenata* (i.e., *Rhopalodia* cf. *musculus* 23vi08.2C.1-GCCT45, *Rhopalodia* sp. 21IV14-4D biarcuate RhopalodiaN2, *Rhopalodia* sp. 13vi08.2B-GCCT21, and *Rhopalodia* sp. 3825.12), and "Remaining E/R" stands for all remaining *Epithemia*/*Rhopalodia* after excluding *E. catenata* and the closely related subset (i.e., " Subset E/R"). Tested topologies include (1) the inclusion of *E. catenata* within genus *Epithemia*; (2) grouping *E. catenata* with a subset of closely related *Epithemia*/*Rhopalodia*, which together form a sister clade to the remaining *Epithemia*/*Rhopalodia*; (3) placing the clade containing *E. catenata* and the subset of closely related *Epithemia*/*Rhopalodia* outside of the rest of *Rhopalodiales*; (4) placing *E. catenata* within Rhopalodiales and sister to all *Epithemia*/*Rhopalodia*; and (5) placing *E. catenata* outside of all Rhopalodiales.

Supplementary References

1. Villar, E. *et al.* The Ocean Gene Atlas: Exploring the biogeography of plankton genes online. *Nucleic Acids Res.* **46,** W289–W295 (2018).
2. Sayers, E. W. *et al.* GenBank. *Nucleic Acids Res.* **49,** D92–D96 (2021).
3. Leinonen, R., Sugawara, H., Shumway, M. & International Nucleotide Sequence Database Collaboration. The sequence read archive. *Nucleic Acids Res.* **39,** D19–21 (2011).
4. Boeuf, D. *et al.* Biological composition and microbial dynamics of sinking particulate organic matter at abyssal depths in the oligotrophic open ocean. *Proc. Natl. Acad. Sci. U.S.A.* **116,** 11824–11832 (2019).
5. Poff, K. E., Leu, A. O., Eppley, J. M., Karl, D. M. & DeLong, E. F. Microbial dynamics of elevated carbon flux in the open ocean's abyss. *Proc. Natl. Acad. Sci. U.S.A.* **118,** e2018269118 (2021).
6. Langlois, R. J., LaRoche, J. & Raab, P. A. Diazotrophic diversity and distribution in the tropical and subtropical Atlantic Ocean. *Appl. Environ. Microb.* **71,** 7910–7919 (2005).
7. Foster, R. A. *et al.* Influence of the Amazon River plume on distributions of free-living and symbiotic cyanobacteria in the western tropical north Atlantic Ocean. *Limnol. Oceanogr.* **52,** 517–532 (2007).
8. Moisander, P. H., Beinart, R. A., Voss, M. & Zehr, J. P. Diversity and abundance of diazotrophic microorganisms in the South China Sea during intermonsoon. *ISME J.* **2,** 954–967 (2008).
9. Kong, L., Jing, H., Kataoka, T., Sun, J. & Liu, H. Phylogenetic diversity and spatio-temporal -distribution of nitrogenase genes (nifH) in the northern South China Sea. *Aquat. Microb. Ecol.* **65,** 15–27 (2011).
10. Bird, C. & Wyman, M. Transcriptionally active heterotrophic diazotrophs are widespread in the upper water column of the Arabian Sea. *FEMS Microbiology Ecology* **84,** 189–200 (2013).
11. Bombar, D., Kubo, K. A. T., Robidart, J., Carter, B. J. & Zehr, J. P. Non-cyanobacterial nifH phylotypes in the North Pacific Subtropical Gyre detected by flow-cytometry cell sorting. *Environmental Microbiology Reports* **5,** 705–715 (2013).
12. Martínez-Pérez, C. *et al.* The small unicellular diazotrophic symbiont, UCYN-A, is a key player in the marine nitrogen cycle. *Nat. Microbiol.* **1,** 1–7 (2016).
13. Chen, M. *et al.* Biogeographic drivers of diazotrophs in the western Pacific Ocean. *Limnol. Oceanogr.* **64,** 1403–1421 (2019).
14. Liu, J. *et al.* Effect of mesoscale eddies on diazotroph community structure and nitrogen fixation rates in the South China Sea. *Regional Studies in Marine Science* **35,** 101106 (2020).
15. Langlois, R. J., Hümmer, D. & LaRoche, J. Abundances and distributions of the dominant nifH phylotypes in the Northern Atlantic Ocean. *Appl. Environ. Microb.* **74,** 1922–1931 (2008).
16. Hashimoto, R., Watai, H., Miyahara, K., Sako, Y. & Yoshida, T. Spatial and temporal variability of unicellular diazotrophic cyanobacteria in the eastern Seto Inland Sea. *Fisheries Science* **82,** 459–471 (2016).

17. Shimodaira, H. & Hasegawa, M. Multiple comparisons of log-likelihoods with applications to phylogenetic inference. *Mol. Biol. Evol.* **16,** 1114–1114 (1999).
18. Shimodaira, H. An approximately unbiased test of phylogenetic tree selection. *Syst Biol* **51,** 492–508 (2002).
19. Katoh, K. & Standley, D. M. MAFFT multiple sequence alignment software version 7: Improvements in performance and usability. *Mol. Biol. Evol.* **30,** 772–780 (2013).
20. Pruesse, E., Peplies, J. & Glöckner, F. O. SINA: accurate high-throughput multiple sequence alignment of ribosomal RNA genes. *Bioinformatics* **28,** 1823–1829 (2012).
