# Supplementary material for: Overlooked and widespread pennate diatom-diazotroph symbioses in the sea
Source: Nat Commun. 2022 Feb 10;13:799. doi: 10.1038/s41467-022-28065-6 (PMC8831587; doi:10.1038/s41467-022-28065-6)
Supplement: Supplementary file 3 — Reporting Summary [file 41467_2022_28065_MOESM3_ESM.pdf]

nature research

Corresponding author(s): Christopher R. Schvarcz

Last updated by author(s): Dec 8, 2021

Reporting Summary

Nature Research wishes to improve the reproducibility of the work that we publish. This form provides structure for consistency and transparency in reporting. For further information on Nature Research policies, see our Editorial Policies and the Editorial Policy Checklist.

Statistics

For all statistical analyses, confirm that the following items are present in the figure legend, table legend, main text, or Methods section.

n/a	Confirmed	
☐	☒	The exact sample size (n) for each experimental group/condition, given as a discrete number and unit of measurement
☐	☒	A statement on whether measurements were taken from distinct samples or whether the same sample was measured repeatedly
☒	☐	The statistical test(s) used AND whether they are one- or two-sided<br>*Only common tests should be described solely by name; describe more complex techniques in the Methods section.*
☒	☐	A description of all covariates tested
☒	☐	A description of any assumptions or corrections, such as tests of normality and adjustment for multiple comparisons
☐	☒	A full description of the statistical parameters including central tendency (e.g. means) or other basic estimates (e.g. regression coefficient) AND variation (e.g. standard deviation) or associated estimates of uncertainty (e.g. confidence intervals)
☒	☐	For null hypothesis testing, the test statistic (e.g. F, t, r) with confidence intervals, effect sizes, degrees of freedom and P value noted<br>*Give P values as exact values whenever suitable.*
☒	☐	For Bayesian analysis, information on the choice of priors and Markov chain Monte Carlo settings
☒	☐	For hierarchical and complex designs, identification of the appropriate level for tests and full reporting of outcomes
☒	☐	Estimates of effect sizes (e.g. Cohen's d, Pearson's r), indicating how they were calculated

Our web collection on statistics for biologists contains articles on many of the points above.

Software and code

Policy information about availability of computer code

Data collection	No software was used for data collection.
Data analysis	Peak Performer RCP (chromatographic analysis), MAFFT v7.453 (nucleotide alignment), trimAl v1.2 (alignment trimming), jModelTest v2.1.10 (selection of nucleotide substitution model for phylogenetics), MrBayes v3.2.7 (phylogenetic inference), RAxML v8.2.12 (phylogenetic inference), IQ-TREE v2.1.3 (phylogenetic tree topology tests), webBLAST (https://blast.ncbi.nlm.nih.gov/)(NCBI non-redundant nucleotide database search), SRA Toolkit v2.10.7 (https://github.com/ncbi/sra-tools)(environmental gene database search), Trimmomatic v0.39 (sediment trap metagenome read quality trimming), BLAST+ suite v2.12.0 (sediment trap metagenome analysis).

For manuscripts utilizing custom algorithms or software that are central to the research but not yet described in published literature, software must be made available to editors and reviewers. We strongly encourage code deposition in a community repository (e.g. GitHub). See the Nature Research guidelines for submitting code & software for further information.

Data

Policy information about availability of data

All manuscripts must include a data availability statement. This statement should provide the following information, where applicable:

- Accession codes, unique identifiers, or web links for publicly available datasets
- A list of figures that have associated raw data
- A description of any restrictions on data availability

Sequences produced for this study have been deposited in GenBank with accession numbers MW562846–MW562894. Analyses were also conducted using data from NCBI's non-redundant nucleotide and protein databases and the following SRA run database accessions: DRR075654–DRR075675, DRR090493–DRR090512, DRR119299–DRR119318, SRR11748760–SRR11748769, SRR11784070–SRR11784101, SRR1994968–SRR1994982, SRR2846720, SRR2846725, SRR2848263–SRR2848264, SRR2848267, SRR2849323, SRR2849339, SRR2849358, SRR2849373, SRR2849384, SRR2849398, SRR2976582–SRR2976583, SRR2988260,

SRR3225470–SRR3225471, SRR3275263–SRR3275264, SRR3502230, SRR3502520–SRR3502528, SRR3898627–SRR3898675, SRR3924383–SRR3924409, SRR5083564–SRR5083575, SRR5693565–SRR5693584, SRR5693645–SRR5693657, SRR576444, SRR576446, SRR576451, SRR576453–SRR576469, SRR5814033–SRR5814187, SRR6057892–SRR6057916, SRR6299285–SRR6299287, SRR6334371–SRR6334373, SRR7142301–SRR7142368, SRR7239923–SRR7239946, SRR7527146–SRR7527159, SRR7632639–SRR7632648, SRR7632653, SRR7632671–SRR7632680, SRR7648270, SRR7648273–SRR7648274, SRR7648284–SRR7648299, SRR7648310, SRR7648320–SRR7648321, SRR7648326–SRR7648327, SRR7648331–SRR7648339, SRR7648341, SRR7648343, SRR7648345–SRR7648350, SRR7668191, SRR7699187–SRR7699216, SRR8104593–SRR8104721, SRR8247196–SRR8247211, SRR8468235–SRR8468237, SRR8468246–SRR8468261, SRR8844064–SRR8844199, SRR9675236.

Field-specific reporting

Please select the one below that is the best fit for your research. If you are not sure, read the appropriate sections before making your selection.

☐ Life sciences ☐ Behavioural & social sciences ☒ Ecological, evolutionary & environmental sciences

For a reference copy of the document with all sections, see nature.com/documents/nr-reporting-summary-flat.pdf

Ecological, evolutionary & environmental sciences study design

All studies must disclose on these points even when the disclosure is negative.

Study description	Two new symbiotic diatom species were described. For each species, multiple isolates were obtained from independent seawater samples, collected on different dates or at different depths. Gene sequencing and light microscopy were performed on all isolates, while scanning electron microscopy and measurements of nitrogen fixation were performed on a single isolate for each species. For each species, diel patterns of nitrogen fixation were determined from two subsamples that were simultaneously collected from the same culture; one subsample was used in the acetylene reduction assay and the other was used in the argon induced dihydrogen production assay. To verify the observed diel patterns in nitrogen fixation, 15N2 assimilation measurements were conducted on triplicate subsamples of each culture, collected at targeted time points. Cell-specific rates of nitrogen fixation were calculated based on the average of triplicate cell concentration measurements. Quantitative PCR was used to quantify the concentration of host and symbiont marker genes over a profile of euphotic zone depths, collected during each of two different months of the same year. For each depth of each sampled month, a single replicate of environmental DNA extract was amplified in four replicate reactions. Standard curves were created using three replicate reactions for each standard.
Research sample	The study focuses on cultures of Epithemia pelagica sp. nov. and Epithemia catena sp. nov. (Rhopalodiaceae). Both species are previously uncharacterized and possess nitrogen-fixing cyanobacterial endosymbionts. These are the first reported marine Rhopalodiaceae to contain such endosymbionts.
Sampling strategy	There was no statistical method used to predetermine the size of the sample used for the metabolic rate measurements shown in Figure 4. The sample size was determined by the detection limit of the gas analyzer (which was 0.03 pmol H2 with a flow rate of 13 ml per min) and the quantities of H2 produced by Epithemia. Therefore, sufficient biomass of the Epithemia species was sub-sampled and analyzed to ensure that the measured signal exceeded the detection limit by at least 10-fold. The number of quantitative PCR reaction replicates were determined by common practices in the field. The single-replicate environmental DNA samples for quantitative PCR were constrained by sample availability. These samples were obtained from a collection of previously archived samples stored at -80 degrees Celsius and represent whole seawater filtered onto 0.02 µm pore size membranes. The sample volume (approx. 2 liters) is the maximum volume that can reasonably be filtered onto these membranes.
Data collection	CRS recorded data related to gene sequencing and microscopy-based morphological measurements. DNA chromatograms were manually inspected to insure gene sequence accuracy, and microscopy-based morphological measurements were determined using scale bars from calibrated instruments. STW and MC recorded all gas-based nitrogen fixation measurements through calibrated chromatographic analysis. QL recorded the quantitative PCR data, and melting curves were analyzed to ensure that non-specific amplification did not occur.
Timing and spatial scale	The environmental sampling was conducted at Station ALOHA, the site of a long-term oceanographic observation program located 100 km north of the Hawaiian Islands. The coordinates for the oceanographic monitoring station are 22.45°N 158°W and the sampling was conducting within an 11 km radius of this location. Samples for Epithemia cultivation were collected on Oct 15, 2014, Feb 21, 2019, May 4, 2019, Jun 13, 2019, and Jul 3, 2019. Samples for qPCR were collected on Jan 16 and Jul 1, 2014. The sampling dates were dictated by the availability of seawater collected by the scientists who run the observing programme.
Data exclusions	No data were excluded from the analyses.
Reproducibility	The main experimental findings are the metabolic rate measurements as shown in Figure 4. All experimental measurements using the preferred H2-based method revealed the same diel pattern. The experiments were repeated on 4-5 occasions and both species of Epithemia demonstrated the same behaviour. Furthermore, the diel pattern was confirmed using two independent measurements: the acetylene reduction assay and stable isotope assimilation. Therefore we are highly confident in the reproducibility of these experimental findings. The ability to cultivate Epithemia from our sampling site (Station ALOHA) was reproduced on two dates for Epithemia catenata and five dates for Epithemia pelagica. The experiment demonstrating the loss of endosymbionts from Epithemia cells grown in nitrogen-replete medium was performed once. We did not attempt to reproduce these results given the long duration of the experiment.
Randomization	Randomization is not relevant to this study as our metabolic rate experiments were conducted over a four month period using multiple generations of Epithemia pelagica and Epithemia catenata. The use of two Epithemia species combined with the multiple lines of investigation negate the need for experimental grouping or randomization. Our objective was to measure the diel patterns of nitrogen fixation and report the consistency of these findings. This objective was achieved.

Blinding	Blinding was not relevant to the analyses conducted in this study, since all raw data was directly recorded and collected by unbiased instruments.

Did the study involve field work? [x] Yes [ ] No

Field work, collection and transport

Field conditions	Temperatures at the sampled depths (5-175 m) at Station ALOHA can range from approximately 18-27 degrees Celsius.
Location	Cultures and environmental samples (used for quantitative PCR) were collected from the subtropical North Pacific Ocean at Station ALOHA (22°45' N, 158°00' W), including water depths of 5, 25, 45, 75, 100, 150, and 175 m.
Access & import/export	Station ALOHA falls within international waters and does not require permits for sample collection.
Disturbance	No disturbance was caused by the study.

Reporting for specific materials, systems and methods

We require information from authors about some types of materials, experimental systems and methods used in many studies. Here, indicate whether each material, system or method listed is relevant to your study. If you are not sure if a list item applies to your research, read the appropriate section before selecting a response.

Materials & experimental systems

n/a	Involved in the study	
[x]	[ ]	Antibodies
[ ]	[x]	Eukaryotic cell lines
[x]	[ ]	Palaeontology and archaeology
[x]	[ ]	Animals and other organisms
[x]	[ ]	Human research participants
[x]	[ ]	Clinical data
[x]	[ ]	Dual use research of concern

Methods

n/a	Involved in the study	
[x]	[ ]	ChIP-seq
[x]	[ ]	Flow cytometry
[x]	[ ]	MRI-based neuroimaging

Eukaryotic cell lines

Policy information about cell lines

Cell line source(s)	The eukaryotic diatom cultures analyzed in this study were isolated from environmental samples collected in the North Pacific Ocean (22°45' N, 158°00' W)
Authentication	The cultures were identified by gene sequencing, and they were confirmed to be unialgal by microscopy observations.
Mycoplasma contamination	Testing for Mycoplasma contamination does not apply to our systems.
Commonly misidentified lines (See ICLAC register)	None.
